# Oxidation Control to Augment Interfacial Charge Transport in Te‐P3HT Hybrid Materials for High Thermoelectric Performance

**DOI:** 10.1002/advs.202400802

**Published:** 2024-07-23

**Authors:** Syed Zulfiqar Hussain Shah, Zhenyu Ding, Zainul Aabdin, Weng Weei Tjiu, Jose Recatala‐Gomez, Haiwen Dai, Xiaoping Yang, Repaka Durga Venkata Maheswar, Gang Wu, Kedar Hippalgaonkar, Iris Nandhakumar, Pawan Kumar

**Affiliations:** ^1^ Institute of Materials Research and Engineering Agency for Science Technology and Research (A*STAR) Singapore 138634 Singapore; ^2^ Department of Chemistry University of Southampton Southampton SO17 1BJ UK; ^3^ High Magnetic Field Laboratory of Anhui Province High Magnetic Field Laboratory HFIPS Chinese Academy of Sciences Hefei 230031 China; ^4^ Science Island Branch of Graduate School University of Science and Technology of China Hefei 230026 China; ^5^ School of Materials Science and Engineering Nanyang Technological University 50 Nanyang Avenue Block N4.1 Singapore 639798 Singapore; ^6^ Institute of High‐Performance Computing Agency for Science Technology and Research (A*STAR) Singapore 138632 Republic of Singapore

**Keywords:** doping, electrical conductivity, hybrid material, microstructural characterization, nanomaterials, P3HT polymer, power factor, Seebeck coefficient, thermoelectric materials, thin film

## Abstract

Organic–inorganic hybrid thermoelectric (TE) materials have attracted tremendous interest for harvesting waste heat energy. Due to their mechanical flexibility, inorganic‐organic hybrid TE materials are considered to be promising candidates for flexible energy harvesting devices. In this work, enhanced TE properties of Tellurium (Te) nanowires (NWs)‐ poly (3‐hexylthiophene‐2, 5‐diyl) (P3HT) hybrid materials are reported by improving the charge transport at interfacial layer mediated via controlled oxidation. A power factor of ≈9.8 µW (mK^2^)^−1^ is obtained at room temperature for oxidized P3HT‐TeNWs hybrid materials, which increases to ≈64.8 µW (mK^2^)^−1^ upon control of TeNWs oxidation. This value is sevenfold higher compared to P3HT‐TeNWs‐based hybrid materials reported in the literature. MD simulation reveals that oxidation‐free TeNWs demonstrate better templating for P3HT polymer compared to oxidized TeNWs. The Kang–Snyder model is used to study the charge transport in these hybrid materials. A large σ_E0_ value is obtained which is related to better templating of P3HT on oxygen‐free TeNWs. This work provides evidence that oxidation control of TeNWs is critical for better interface‐driven charge transport, which enhances the thermoelectric properties of TeNWs‐P3HT hybrid materials. This work provides a new avenue to improve the thermoelectric properties of a new class of hybrid thermoelectric materials.

## Graphical abstract

This study explores the impact of Tellurium nanowires (TeNWs) oxidation on thermoelectric properties within a P3HT‐TeNWs hybrid system. A fivefold enhancement, in power factor is observed through controlling the oxidation. Specifically, the absence of oxidation on the TeNWs surface exhibits superior templating capabilities for P3HT polymer compared to oxidized TeNWs, which enhances the mobility of the hybrid system.

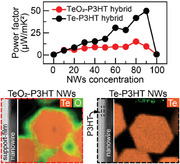

## 1 Introduction

Thermoelectric (TE) devices have the ability to directly convert heat into electricity, without the need for moving parts.^[^ ^1^, ^2^ ^]^ The efficiency of a TE device is related to the dimensionless figure of merit (*zT*) which is defined as $zT=\frac{S^{2}\sigma T}{\kappa }$, where *S* is the Seebeck coefficient, σ is the electrical conductivity, *T* is the absolute temperature, *κ* is the thermal conductivity, and *S* ^2^σ is the power factor (PF) of the TE material.^[^ ^2^ ^]^ A low *κ* and high PF are hence required to achieve better‐performing TE materials. Due to the strong interdependence of transport parameters (*S, σ*, and *κ*) optimizing the TE performance is a complex task. Nanostructuring of inorganic materials is a promising strategy to control these interdependencies and significant improvements in *zT* of inorganic thermoelectric materials have been achieved recently.^[^ ^2^, ^3^ ^]^ Substantial research endeavors have been dedicated to incorporating these nanostructured inorganic materials into the matrix ofconducting polymers in recent years.^[^ ^4^, ^5^, ^6^, ^7^, ^8^, ^9^, ^10^, ^11^, ^12^, ^13^, ^14^, ^15^, ^16^ ^]^ For example, Wang et al.,^[^ ^5^ ^]^ synthesized a polyaniline (PANI)‐Te composite that exhibited a Seebeck coefficient of 102 µV K^−1^, electrical conductivity (σ) of 102 S cm^−1^, and a power factor (PF) of 105 µW (m K^2^)^−1^. Sahu reported a PF of 145 µW (mK^2^)^−1^ for poly(3,4‐ethylenedioxythiophene):poly(styrenesulfonate) (PEDOT:PSS)/TeNWs,^[^ ^18^ ^]^ and Gordon achieved a PF of 130 µW (m K^2^)^−1^ at room temperature using PEDOT:PSS/TeNWs.^[^ ^19^ ^]^ Nevertheless, numerous polymer/inorganic composites, primarily employing conducting polymers, have been recorded to demonstrate a significant thermoelectric power factor exceeding 100 µW (mK^2^)^−1^.^[^ ^5^, ^12^, ^17^, ^18^, ^19^, ^20^ ^]^

Conversely, polymer/inorganic composites based on P3HT have been explored to a limited extent so far. For instance, Ming He et al.,^[^ ^4^ ^]^ incorporated Bi_2_Te_3_ nanowires (NWs) into P3HT, yielding a composite with a power factor of 13.6 µW (m K^2^)^−1^. Liang studied the P3HT/TeNWs composite system, achieving an electrical conductivity of 21.3 S cm^−1^, Seebeck coefficient of 67.1 µV K^−1^, and a power factor of ≈9.59 µW (mK^2^)^−1^ with optimal 30% FeCl_3_ doping.^[^ ^11^ ^]^ Interfacial transport, structural/morphological effects, and modifications to the energy dependence of carrier scattering (energy filtering) to improve electronic and thermoelectric performance have been suggested to explain the charge transport in these hybrid materials.^[^ ^4^, ^11^, ^21^, ^22^, ^23^ ^]^ Later Kumar et al.,^[^ ^24^ ^]^ demonstrated that the high thermoelectric performance observed in these complex hybrid systems can be explained by physical interfacial interactions between the inorganic and organic components which enhance the Seebeck coefficient and carrier mobility in these hybrid materials. Experimentally a highly ordered morphology of conducting polymers has been demonstrated through interfacial interactions with inorganic nanostructures at the interface which give rise to a high electrically conductive path for charge carriers.^[^ ^22^, ^24^, ^37^, ^38^ ^]^ Besides, It has been demonstrated that these inorganic nanostructures are prone to oxidation which potentially affects the thermoelectric properties of these hybrid materials.^[^ ^25^ ^]^

In this work, we have improved the interfacial interaction of Te‐P3HT hybrid materials through controlled oxidation. The findings highlight a substantial improvement in the power factor of the P3HT‐TeNWs hybrid nanocomposites. We demonstrate that precise oxidation control during the synthesis of inorganic TeNWs and the preparation of the hybrid film is crucial for improving the templating of P3HT along the TeNWs, and ultimately improving the interfacial transport within the hybrid material. The resulting P3HT‐TeNWs thermoelectric nanocomposites exhibited the highest power factor of 64.8 µW (mK^2^)^−1^ at room temperature. This achievement represents a sevenfold increase in comparison to previously documented values in the literature (Figure S11, Supporting Information).^[^ ^11^ ^]^ Due to the inherently low thermal conductivity of these hybrid materials.^[^ ^4^, ^5^, ^17^ ^]^ The optimization efforts focused on enhancing the power factor of the P3HT‐TeNWs composite hybrid material (instead of zT) and the interfacial layer by controlling the oxidation level to optimize the charge transport properties in these systems.

## 2 Results and Discussion

**Figure 1:**
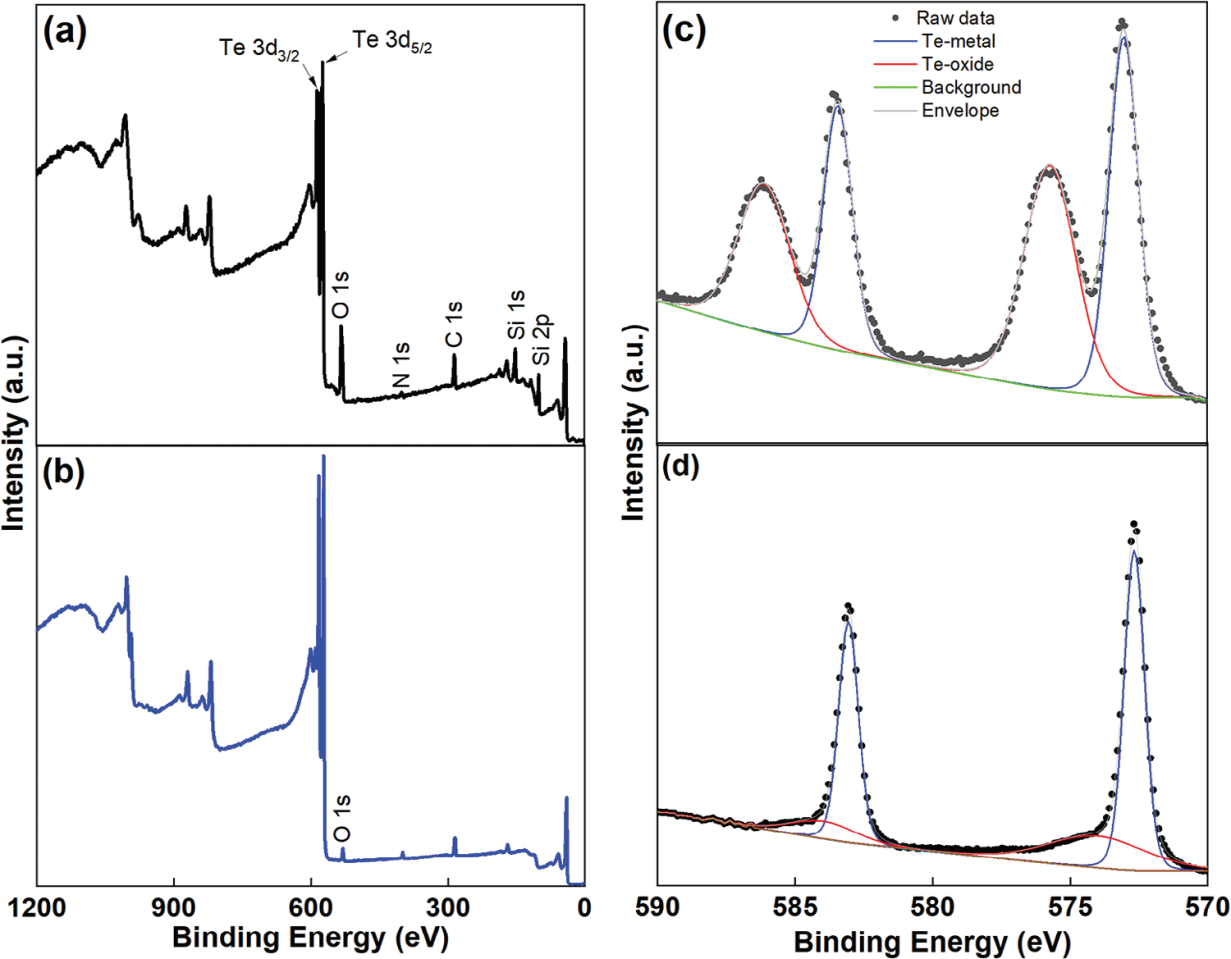
High‐resolution X‐ray photoelectron spectroscopy (XPS) spectra of oxidized TeNWs films and oxidation control TeNWs: Survey scan and XPS core level spectra of Te 3d of highly oxidized TeNWs a–c), and oxidation controlled TeNWs b–d), respectively. A lower oxidation level was observed in oxidation‐controlled TeNWs compared to oxidized TeNWs.

P3HT‐TeNWs nanocomposite hybrid films with varying TeNWs to P3HT ratios (10–90 wt.%) were prepared by dispersing the P3HT‐TeNWs powder in chloroform followed by drop casting. Optical micrographs of the resulting films show a uniform thermoelectric thin film (Figure S1, Supporting Information). The thickness of P3HT‐TeNWs nanocomposite films was measured by surface profilometry and varied from 5 ± 0.87 to 12 ± 2.82 µm with lower concentrations of Te nanowires yielding thicker films. To identify the elemental species and measure the oxidation level within the fabricated thin films, X‐ray photoelectron spectroscopy (XPS) was performed, and the results are shown in **Figure** **1**. The XPS survey spectra in Figure 1a,b (up to 1200 eV) revealed the presence of tellurium, carbon, and oxygen. All spectra were calibrated with respect to the carbon C 1s peak at 284.8 eV. Core‐level peaks corresponding to Te 3d, C 1s, and O 1s were observed in thin films of oxidized tellurium and oxidation‐controlled tellurium nanowires. The O 1s peak ≈530 eV corresponds to TeO_2_ which originates from an oxidized TeNWs surface (see high resolution O 1s spectra in Figure S9b, Supporting Information).^[^ ^27^ ^]^ Peaks centered at a binding energy of 573.07 and 583.3 eV are attributed to the Te 3d_5/2_ and Te 3d_3/2_ core levels respectively, indicating that tellurium is in its metallic state.^[^ ^28^ ^]^ In Figure 1c there are observable peaks at 576 and 586 eV that indicate an oxidized surface of tellurium NWs with 45 atomic percent oxygen content within 5–10 nm depth of the film.^[^ ^29^ ^]^ Figure 1d indicates that the oxygen content is reduced to 12 atomic percent at the surface of the NWs prepared by oxidation control within 5–10 nm depth of the film. The oxygen atomic percentage is determined from the survey spectra and subsequently compared with the high‐resolution Te 3d spectra, showing matching values in both scenarios. In addition, we computed the ratio of Te 3d_5/2_‐oxide to Te 3d_5/2_‐metal, resulting in a decrease from 0.8 to 0.2 for oxidized‐TeNWs and oxidation‐controlled‐TeNWs, respectively.

The peak ≈400.5 eV is identified as the sole nitrogen peak, possibly resulting from the use of hydrazine hydrate as a reducing agent during synthesis. Additionally, as the samples were prepared on Si substrates, the characteristic peaks at binding energies ≈100.01 and 151.09 eV are attributed to Si 2p and Si 1s, respectively.^[^ ^36^ ^]^

**Figure 2:**
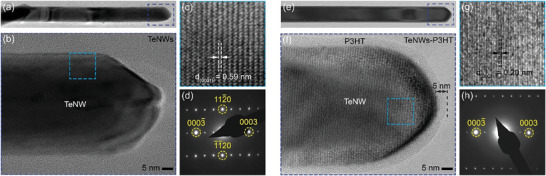
High‐resolution TEM and selected area electron diffraction (SAED) of individual TeNW and P3HT‐coated TeNW. High‐resolution TEM images showing the morphology of individual NWs of a–c) TeNWs and e–g) P3HT‐TeNWs with a uniform conformal coating of the amorphous polymer (P3HT) layer on the surface of the nanowire. Selected area electron diffraction pattern of a single d) TeNWs and h) P3HT‐TeNWs.

Detailed microstructural characterizations were performed by using a Scanning Electron Microscope (SEM) and Transmission Electron Microscope (TEM) to investigate the distribution of Te nanowires within the P3HT polymer matrix and the uniformity of the polymer coating on individual nanowires (Figures S2 and S3, Supporting Information). A homogenous dispersion of TeNWs within P3HT matrix is observed for all concentrations in the SEM images (Figure S2b, Supporting Information). TEM images acquired from individual TeNWs and P3HT‐TeNWs nanowires show that the microstructures of both TeNWs and P3HT‐TeNWs are identical, with P3HT‐TeNWs having a uniform conformal coating of the polymer (P3HT) layer on the surface of the nanowires (**Figure** **2**a,b,e,f). The thickness of the coating is measured to be ≈5 nm. High‐resolution TEM (HR‐TEM) images combined with selected area electron diffraction (SAED) reveal that the nanowires are single crystalline, and their *c*‐axis is always aligned along the length of the nanowires with an interplanar spacing of ≈0.59 nm that corresponds to the (001) reflection. Furthermore, the nanowires have a hexagonal shape, bounded by six (100) facets with measured interplanar spacing of ≈0.39 nm along all facets, as seen in the cross‐section HR‐TEM images (Figures S4 and S5, Supporting Information). Low‐magnification TEM images show that the TeNWs are quite uniform in diameters (≈65 ± 12 nm), although the nanowire length varies from a few hundred nanometers to several micrometers (≈500–4000 nm).

**Figure 3:**
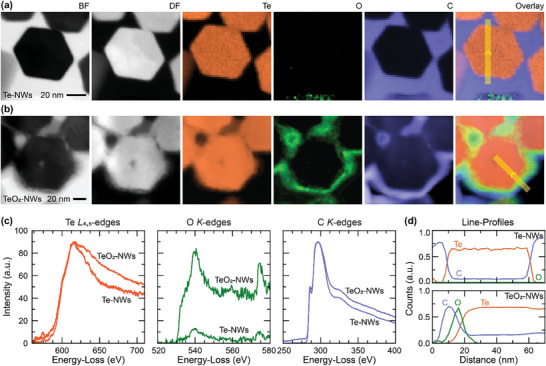
Scanning Transmission Electron Microscope (STEM) imaging and Electron Energy Loss Spectroscopy (EELS) mapping to measure the oxygen content in oxidized TeNWs and oxidation‐controlled TeNWs. Bright‐field (BF), dark‐field (DF) STEM images, corresponding elemental (Te, O, C) EELS maps, and overlay (Te + O + C) maps of the a) Te‐NWs and b) TeO_2_‐NWs in cross‐section. c) Te *L*_4,5_‐edges, O *K*‐edges, and C *K*‐edges for the two cases extracted from the spectral image. d) Averaged and normalized line profile across the nanowire marked with the yellow strips in (a,b). No oxidation was observed on the surface of controlled oxidation TeNWs while a thin layer of oxidation was seen on oxidized TeNWs.

For optimal performance of the hybrid materials, the nanowires surface should be free from any impurities and oxide. Therefore, we performed Scanning Transmission Electron Microscope (STEM) imaging and Electron Energy Loss Spectroscopy (EELS) mapping to measure the oxygen content at the nanoscale. For both techniques, we used a probe size of ≈1 nm. No oxygen traces were found in the Te‐NWs (**Figure** **3**a), whereas the nanowires in Figure 3b clearly show the presence of oxygen around the edges of the nanowires (green color maps) and an overall increase in the oxygen signal in the spectra (Figure 3c). This indicates surface oxidation of the Te‐NWs (hereafter we will refer to them as TeO_2_‐NWs). A closer look at the overlay maps and line profiles obtained for the nanowire presented in Figure 3d reveals that the nanowire core is free from oxidation for both Te‐NWs and TeO_2_‐NWs, whilst only the surface of the nanowire is oxidized for TeO_2_‐NWs. It is worthwhile to note that the Carbon (C) (blue maps in both cases) signal present in the EELS data does not originate from the synthesis process of the nanowires, instead, C was deposited as part of the FIB cross‐section sample preparation to protect the nanowire surface from beam damage during FIB cutting. High‐resolution TEM images of the oxide layer formed at the nanowire surface or at the interface of two nanowires show that the oxide layer is crystalline in nature (Figure S6c, Supporting Information). Interestingly, the oxidation phenomena not only affect the nanowire surface but also introduce a lot of defects inside the Te core as seen in the TEM/STEM images presented in Figures S6–S8 (Supporting Information). This is most likely due to the structural changes that occur at the Te nanowire surface due to oxide formation. It is worthwhile to note that Te exhibits a hexagonal crystal structure (with lattice constant a = b = 4.19 Å, c = 5.98 Å), whereas TeO_2_ exhibits a tetragonal crystal structure (with lattice constant a = b = 5.41 Å, c = 13.20 Å).^[^ ^27^, ^30^ ^]^ It is also revealed in X‐ray diffraction patterns in Figure S9 (Supporting Information).

**Figure 4:**
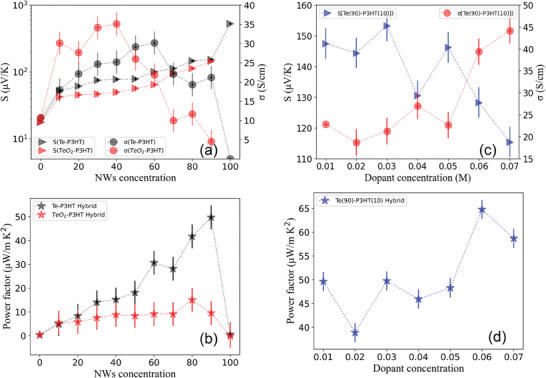
Thermoelectric properties of P3HT based inorganic (TeNWs & TeO_2_NWs) hybrid systems as a function of TeNWs content and dopant concentration. a) Electrical conductivity and Seebeck coefficient & b) power factor of 0.03 m FeCl_3_ doped hybrid films as a function of weight ratio of NWs in the P3HT matrix range from 0 to 100 wt.%. c) Electrical conductivity and Seebeck coefficient & d) power factor as a function of molar concentration of FeCl_3_ dopant for P3HT‐TeNWs hybrid films with 90 wt.% TeNWs concentration. The vertical error bars represent the standard deviation of multiple measurements with respect to average values for each sample.

The hybrid films comprising different weight percent (wt.%) ratios of TeNWs and P3HT were immersed in 0.03 m FeCl_3_ solution for doping before measuring their TE transport properties. The Seebeck coefficient and electrical conductivity of P3HT‐TeNWs nanocomposite hybrid films were measured by standard methods as detailed in the Experimental Section. **Figure** **4**a presents the thermoelectric transport properties of P3HT‐TeNWs hybrid and P3HT‐TeO_2_NWs hybrid films as a function of varying concentrations (by weight) of NWs. For the P3HT‐TeNWs hybrid system, it can be observed that with increasing concentration of TeNWs, the Seebeck coefficient increases monotonically (cf, black arrows) in Figure 4a. The Seebeck coefficient changed from 17.8 ± 0.81 to 528.8 ± 4.23 µV K^−1^ by increasing the TeNWs concentration from 0% to 100%. The electrical conductivity also increases initially with increasing TeNWs content (cf. black circles) in Figure 4a. It increases from ≈11 to ≈34 S cm^−1^ when the TeNWs content is increased from 0 to 60 wt.%, and then remains constant at ≈20 S cm^−1^ (70%–90% TeNWs) before declining to ≈0.02 S cm^−1^ (100% TeNWs). A similar trend has been observed in TeNW‐PEDOT: PSS system.^[^ ^22^ ^]^ The trends for the P3HT‐TeO_2_NWs hybrid system are similar where conductivity (red circles) initially increases with NWs content and reaches ≈35 S cm^−1^ for 30% NWs content. Then, it drops as the NW content increases further up to ≈5 S cm^−1^ for 90% NWs content. On the other hand, the Seebeck coefficient (red arrows) increases monotonically with increasing NW content. Figure 4b shows the power factor as a function of NW content for P3HT‐TeNWs (black stars) and P3HT‐TeO_2_NWs (red stars) hybrid systems. While the power factor for P3HT‐TeNWs increases from 0.34 µW (mK^2^)^−1^ (0% TeNWs) to 49.8 µW (mK^2^)^−1^ (90% TeNWs) for doped P3HT‐TeNWs hybrid system, it increases from 0.34 µW (mK^2^)^−1^ (0% TeNWs) to ≈15 µW (mK^2^)^−1^ (80% TeO_2_NWs) for P3HT‐TeO_2_NWs hybrid films.

The P3HT‐TeNWs hybrid sample with the highest power factor (90 wt.% TeNWs) was selected for further optimization of the TE properties by tuning the doping level. Seven hybrid films were fabricated and immersed in varying molar concentrations (0.01–0.07 m) of FeCl_3_‐acetonitrile solutions to analyze TE trends in P3HT‐TeNWs hybrid films and are shown in Figure 4c,d. Doping of the hybrid films with different concentrations of FeCl_3_ introduces more charge carriers at the P3HT‐TeNWs interface which contributes to enhancing the electrical conductivity of the hybrid films. On the other hand, increasing the carrier concentration leads to a decrease in the Seebeck coefficient (Figure S10, Supporting Information).^[^ ^31^, ^32^ ^]^ Figure 4c shows that the Seebeck coefficient decreases from 147.48 to 115.3 µV K^−1^ when the molar concentration of FeCl_3_ increases from 0.01–0.07 m. The electrical conductivity changed from 22.8 to 44.2 S cm^−1^ by changing the dopant concentration from 0.01–0.07 m. The power factor of the hybrid films also increases with increasing FeCl_3_ concentration reaching a maximum value of 64.8 µW (mK^2^)^−1^ at 0.06 m as shown in Figure 4d. This suggests that the incorporation of nanowires within a conducting polymer matrix could effectively enhance the power factor of nanocomposite hybrid materials at the optimum doping level using a suitable dopant concentration.

Next, we studied the charge transport in the P3HT‐TeNWs hybrid system (90 wt.% TeNWs‐10% P3HT) using the Boltzmann Transport framework as developed for conducting polymers by Kang and Snyder (Kang–Snyder Model).^[^ ^26^ ^]^ According to the Kang–Snyder model, the energy‐dependent conductivity σ_*E*_(*E*,*T*) can be expressed as:

(1) $$\sigma_{E}\left(E,T\right)=\sigma_{E_{0}}\left(T\right){\left(\frac{E-E_{t}}{k_{B}T}\right)}^{s}$$

where, E_t_ is the transport edge (energy) below which conductivity has no contribution even at finite temperature, “s” is the energy‐dependent scattering parameter, and “σ_E0_” is the energy‐independent transport parameter to model the TE transport of conducting polymers over a large range of conductivities. k_B_ is the Boltzmann constant. The total conductivity is given by:

(2) $$\sigma =\int_{0}^{\infty }\sigma_{E}\left(E,T\right)\left(-\frac{\partial f}{\partial E}\right)\mathrm{dE}$$

By inserting Equation (1) into Equation (2), and integrating by parts, the total conductivity can be expressed as:

(3) $$\sigma =\sigma_{E_{0}}\left(T\right)\times sF_{s-1}\left(\eta \right)$$

Where $\eta =\frac{E_{F}-E_{t}}{k_{B}T}$ is the reduced chemical potential and F is the Fermi integral. The corresponding Seebeck coefficient (S) can be expressed as:

(4) $$S=\frac{k_{B}}{e}\left[\frac{\left(s+1\right)F_{s}\left(\eta \right)}{sF_{s-1}\left(\eta \right)}-\eta \right]$$

**Figure 5:**
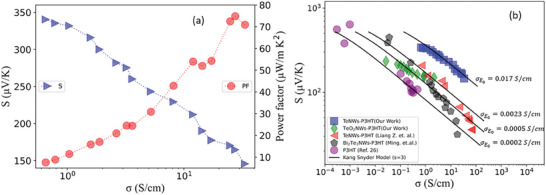
The Kang–Snyder charge transport (CT) model applied to P3HT‐based nanocomposite hybrid systems. a) The experimental Seebeck (S) versus electrical conductivity (σ) data of 0.06 m FeCl_3_ doped P3HT‐TeNWs hybrid system b) The electrical conductivity versus Seebeck coefficient data of P3HT‐TeNWs (blue square) from this work, TeO_2_NWs‐P3HT (green diamond) from this work, P3HT‐TeNWs (red triangles, Liang et. al.,), Bi_2_Te_3_‐P3HT (grey pentagons, Ming et. al.,), and pure P3HT (purple circles, Ref. [26]). Our experimental data lies on the energy‐dependent scattering parameter s = 3 curves with energy‐independent transport parameter σ_E0_ = 0.017 S cm^−1^.

The reduced chemical potential (*η*) is determined by using the experimental values of the Seebeck coefficient for a specific value of the energy‐dependent parameter *s*. **Figure** **5**a shows the Seebeck coefficient as a function of conductivity for the P3HT‐TeNWs hybrid sample (90%TeNWs‐10% P3HT). The data was collected by performing de‐doping experiments. During the de‐doping process, the sample was heat treated at 50 ⁰C to slowly remove the dopant which resulted in a drop of the electrical conductivity. As expected, the Seebeck increases with decreasing conductivity. The power factor was observed to drop gradually with decreasing electrical conductivity.

Figure 5b shows the Seebeck coefficient as a function of the electrical conductivity of P3HT‐TeNWs (blue squares, this work), P3HT‐TeO_2_NWs (green diamond, this work), P3HT‐TeNWs (red triangles, Liang et al.),^[^ ^11^ ^]^ pure P3HT (purple circles, Kang and Snyder),^[^ ^26^ ^]^ Bi_2_Te_3_‐P3HT (grey pentagons, Ming H. et al.).^[^ ^4^ ^]^ The Kang–Snyder model was applied to these systems. As can be seen in Figure 5b the experimental data for all the different P3HT‐based hybrid materials exhibit an energy‐dependent scattering parameter of s = 3. For our P3HT‐TeNWs hybrid system, σ_E0_ was found to be 0.017 S cm^−1^, which is one order higher compared to the P3HT‐TeNWs hybrid system reported in the literature.

**Figure 6:**
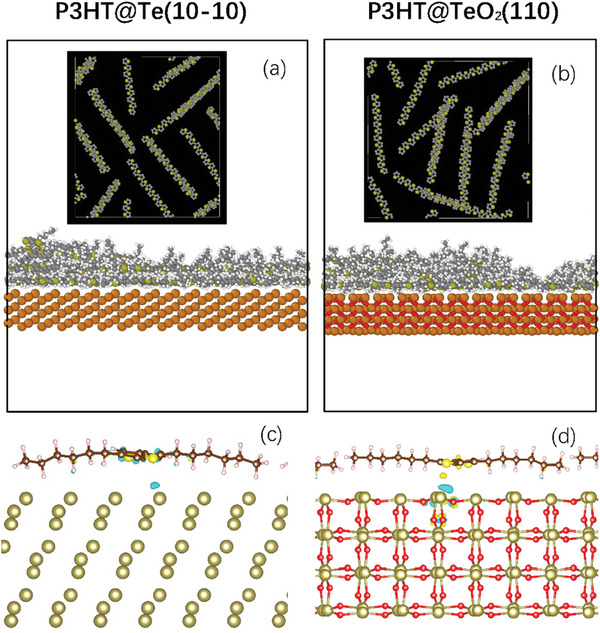
MD simulation of P3HT polymer morphology and alignment at the organic–inorganic interface on Te and TeO_2_ surface. Here, the final polymer structures are depicted after simulated annealing of five chains of P3HT on a) Te (hexagonal) and b) TeO_2_ (tetragonal) surfaces. There is a high concentration of S atoms of P3HT observed at 3–5 Å from the nanowire surfaces, suggestive of highly ordered and aligned P3HT chains at the organic–inorganic interface. Although alignment occurs, the self‐assembly of chains is reduced on TeO_2_ compared to the Te surface. c) DFT calculations reveal electronic effects at the organic–inorganic interface. Electron transfer from the Te surface to P3HT chains is monitored by an increase of electron density (yellow) on P3HT and at the interface and a decrease of electron density (cyan) at the Te phase. d) Electron transfer from TeO_2_ to P3HT. The iso‐values in (c) and (d) are 10^−3^ and 2.5 × 10^−3^ Å^−3^, respectively. The amount of charge transfer is more significant in the case of TeO_2_ substrate.

In order to explain the higher value of σ_E0_ and the conductivity trend, we performed molecular dynamics (MD) simulations to understand the ordering of P3HT polymer on both Te and TeO_2_ surfaces. Whilst P3HT aligns preferentially on a pristine Te surface, a different preferential orientation of P3HT was observed on a TeO_2_ surface (Figure S16, Supporting Information). These findings reveal that the templating is more effective on the Te surface compared to a TeO_2_ surface as shown in **Figure** **6**a,b. To initialize the MD simulations, we assumed TeO_2_ to adopt a crystalline tetragonal structure, as observed experimentally in TEM. However, it is important to note that while the interface between Te core and TeO_2_ exhibits a crystalline nature, the regions further from the interface display an amorphous oxide (Figure S6c, Supporting Information). As a result, we anticipate that the polymer templating will be even less efficient for TeO_2_ nanowire, leading to a lower value of σ_E0_, as observed in Figure 5.

The highest value of σ_E0_ is observed for Te‐P3HT hybrid material, primarily due to a better templating of P3HT on Te surface, as supported by MD simulations. Figure 6c,d demonstrates the charge transfer from the inorganic surface to the first layer of doped P3HT polymer chains. Here, a positive quantity represents electron transfer from the inorganic surface to the organic doped P3HT chains (i.e., hole transfer from the organic P3HT chain to the inorganic surface (cyan color)). In both cases, this charge transfer induces a de‐doping effect, leading to a reduction of holes in the p‐type P3HT chains. This de‐doping effect is more pronounced for doped P3HT on the TeO_2_ surface compared to doped P3HT on the Te surface. However, it is essential to note that the charge transfer and de‐doping effect are only observed for the first two layers of P3HT chains and diminish for greater distances from the inorganic surface. A more detailed analysis of the mechanism of charge transfer can be found in the Supporting Information. This charge transfer phenomenon plays a crucial role in understanding the thermoelectric trends in these hybrid systems.

We now discuss the Seebeck and conductivity trend as a function of Te content. Interestingly, while the Seebeck coefficient increases monotonically, the electrical conductivity of the films exhibits a peak with increasing Te in our hybrid films. Standard binary models in literature are not able to explain these non‐monotonic trends of Seebeck and electrical conductivity in our hybrid films (Figure S13, Supporting Information).^[^ ^33^ ^]^ N. E. Coates later proposed a series‐connected model for these hybrid materials that includes a highly conductive layer between the Te nanowires and polymer matrix can accurately describe the Seebeck and electrical conductivity trend in these hybrid materials.^[^ ^22^ ^]^ We have further shown that a high conductive interfacial layer occurs due to a strong templating effect at the interface using MD simulation. Therefore, the total conductivity of TeNW‐P3HT hybrid can be written as:

(5) $$\sigma_{eff}=x_{Te}\;\sigma_{Te}+x_{interface}\sigma_{interface}+x_{P3HT}\sigma_{P3HT}$$

where σ_eff_ is the conductivity of the hybrid material, *x_Te_* is the fraction of TeNW, *σ_Te_* is the conductivity of the Te nanowires, *x_s_* is the fraction of the interfacial component, *σ_s_* is the conductivity of the interfacial layer, *x*_P3HT_ is the P3HT matrix fraction, and *σ_P3HT_* is the matrix conductivity. Seebeck coefficient can be written as:

(6) $$S=\frac{x_{Te}S_{Te}\sigma_{Te}+x_{interface}S_{interface}\sigma_{interfeace}+x_{P3HT}S_{P3HT}\sigma_{P3HT}}{\sigma_{eff}}$$

At lower TeNW content (<10%), P3HT matrix dominates the transport. Therefore, a similar value of power factor is observed for P3HT‐TeNW and P3HT‐TeO_2_NW hybrid materials. As the nanowire content increases, both the templating effect and charge transfer between NWs and P3HT interface increase. While templating gives rise to higher mobility at the interface, charge transfer at the interface introduces de‐doping which enhances the Seebeck coefficient of the interface layer. Both these factor controls the conductivity and Seebeck coefficient trend in P3HT‐TeNW and P3HT‐TeO_2_NW hybrid materials as discussed in **Table** **1**.^[^ ^22^, ^24^ ^]^

**Table 1:** Summary of physical phenomena contributing to thermoelectric trends at P3HT‐TeNWs and P3HT‐TeO_2_NWs interface.

	Templating effect	De‐doping due to Charge transfer at interface	Seebeck and conductivity	Discussion
P3HT	X	X	X	
P3HT‐TeNWs (10–60%)	µ(↑)	*n*_de‐doping_(↓) *S*_de‐doping_(↑)	$\begin{array}{c} \sigma =n_{de-doping}\left(↓\right)\times e\times \mu \left(↑\right)\\ S\left(↑\right)\propto \frac{1}{n_{de-doping}\left(↓\right)} \end{array}$	Conductivity increases due to Strong templating compared to a reduction in carrier concentration; Seebeck coefficient increases due to charge transfer induced de‐doping at the interface. Strong templating takes over carrier concentration reduction to improve the conductivity
P3HT‐TeNWs (70–90%)	µ(↑)	*n*_de‐doping_(↓) *S*_de‐doping_(↑)	$\begin{array}{c} \sigma =n_{de-doping}\left(↓\right)\times e\times \mu \left(↑\right)\\ S\left(↑\right)\propto \frac{1}{n_{de-doping}\left(↓\right)} \end{array}$	Conductivity stays constant due to comparable *n* reduction rate and templating effect; Seebeck increases due to charge transfer induced de‐doping.
P3HT‐TeO_2_NWs (10–40%)	µ(↑)	*n*_de‐doping_(↓) *S*_de‐doping_(↑)	$\begin{array}{c} \sigma =n_{de-doping}\left(↓\right)\times e\times \mu \left(↑\right)\\ S\left(↑\right)\propto \frac{1}{n_{de-doping}\left(↓\right)} \end{array}$	Conductivity increases due to templating compared to the reduction in carrier concentration; Seebeck coefficient increases due to charge transfer induced de‐doping at the interface. Templating takes over carrier concentration reduction to improve the conductivity
P3HT‐TeO_2_NWs (40–90%)	µ(↑)	*n*_de‐doping_(↓) *S*_de‐doping_(↑)	$\begin{array}{c} \sigma =n_{de-doping}\left(↓\right)\left(\right)\times e\times \mu \left(↑\right)\\ S\left(↑\right)\propto \frac{1}{n_{de-doping}\left(↓\right)} \end{array}$	Conductivity drops due to a stronger *n* reduction rate as observed in the charge transfer section in DFT and weak templating effect; Seebeck increases due to charge transfer induced de‐doping.

## 3 Conclusion

In summary, we have demonstrated that the TE properties in P3HT‐TeNWs hybrid systems can be significantly enhanced by controlling the oxidation of the Te NWs during the synthesis, followed by a strong templating effect. TEM study revealed amorphous oxidation on the Te NW surface for oxidized samples whilst for oxidation‐controlled samples, no oxidation was detected. MD simulation demonstrated a strong uni‐directional templating effect of P3HT on a Te surface compared to a TeO_2_ surface which resulted in a multi‐directional templating of P3HT. DFT calculations reveal strong de‐doping effects at the TeO_2_‐P3HT interface compared to a Te‐P3HT interface. To the best of our knowledge, a power factor of 64.8 µW (mK^2^)^−1^ is the highest value reported for the P3HT‐TeNWs hybrid system to date. Our work provides a new direction to improve TE properties, and in general, charge transport in hybrid TE materials.

## 4 Experimental Section

### Reagents

Ethylene glycol (>99% EG, Sigma–Aldrich, anhydrous), polyvinylpyrrolidone (PVP‐K30, M.W. ≈40000, Fluka), potassium hydroxide (≥ 85% KOH basis, pellets, white, Merck), tellurium dioxide (99.995% TeO_2_, Aldrich), hydrazine hydrate (N_2_H_4_ 50%–60%, Sigma–Aldrich), Poly(3‐hexylthiophene‐2,5‐diyl) (M.W. ≈50–70 kDa, regioregular electronic grade, Rieke Metals), iron trichloride (≥99.99%, anhydrous, powder, Aldrich), acetonitrile (98%, Sigma–Aldrich), chloroform (>99%, anhydrous, Sigma–Aldrich), were used as supplied without further purification.

### Synthesis of Inorganic–Organic Nanocomposites

The inorganic nanostructures of tellurium nanowires (TeNWs) were synthesized via an aqueous solution route following established literature protocols.^[^ ^34^ ^]^ Poly (3‐hexylthiophene) P3HT was dissolved in chloroform to obtain a stock solution (10 mg mL^−1^) followed by magnetic stirring at 40 °C on a hotplate for 30 min. The desired amount of polymer solution was added to various weight percentages (10%–90%) of tellurium nanowires (TeNWs). These solutions were power sonicated for 60 min in pulse mode (15 sec ON and 5 sec OFF) to obtain a homogenous dispersion before being drop‐casted onto Si and quartz substrates.

### Thin Film Fabrication

The solutions were drop cast onto round quartz substrates of 20 mm diameters for thermoelectric (TE) transport properties measurements. For X‐ray diffraction (XRD), scanning electron microscopy (SEM), and X‐ray photoelectron spectroscopy (XPS) analysis, films were drop cast onto (1 × 1) cm^2^ Si substrates at 70 °C on a hotplate. The drop casted films were annealed at 100 °C overnight inside a nitrogen‐filled glovebox to remove any solvent present. For doping the fabricated hybrid films, FeCl_3_ was dissolved in acetonitrile at various molar concentrations of FeCl_3_. The hybrid films were immersed into the FeCl_3_ solution followed by rapid drying to maintain the uniformity of the films. The films were not exposed to air at any point. Prior to drop casting, all substrates were cleaned by sequential sonication in acetone and isopropanol for 10 min each, followed by UV‐ozone exposure at 100 °C for 10 min.

### Characterization

Focused ion beam (FIB) cross‐sectioning of the drop‐casted nanowires was carried out on a Zeiss Crossbeam 540 equipped with carbon GIS and Ga ion source. Three amorphous carbon layers as first deposited onto the nanowires, including a 20 nm layer via pre‐sputtering outside FIB chamber, a 50 nm layer via e‐beam, and a 700 nm layer via ion‐beam. Ga ion‐beam was used for the FIB lamella preparation. The sample was finally fine‐polished using a 30 kV 50 pA probe and cleaned using a 5 kV 10 pA probe. The X‐ray diffraction patterns were collected using a Bruker D8 Advanced X‐ray powder diffractometer with Cu Kα radiation at room temperature. X‐ray photoelectron spectroscopy (XPS) was performed using an AXIS Supra spectrometer (Kratos Analytical, UK) equipped with a hemispherical analyzer and a monochromatic Al Kα source (1487 eV) operating at 15 mA and 15 kV. The XPS spectra were acquired over an analysis area of 700 × 300 µm^2^ at a take‐off angle of 90⁰. A pass energy of 160 and 20 eV was used for survey and high‐resolution scans, respectively. Charge compensation was achieved by using low‐energy electron flooding. Deconvolution of the raw data was performed using the Casa XPS software. Scanning electron microscopy (SEM) imaging was performed on a JEOL JSM 7800F Prime‐scanning electron microscope (SEM) at an operating voltage of 5 keV. To perform Transmission electron microscopy (TEM) analysis, samples were prepared by drop‐casting a 20 µL solution on a standard 3 mm copper mesh grid with a continuous lacey carbon coated film (Cat. No. 3830C‐CF, SPI Supplies, West Chester, USA). Suspension solutions were prepared by mixing 50 µg of powder containing the nanowires (TeNWs or P3HT‐TeNWs) into 1 mL ACS‐grade water (Cat. No. 320072, Sigma–Aldrich Co. LLC, St. Louis, USA). The suspension solutions were thoroughly mixed using a vortex mixer (Scientific Industries Inc., New York, USA) for 2 min prior to dip casting on the TEM grid to uniformly disperse the nanoparticles in the solution. Grids were naturally dried for ≈1 h prior to loading inside the TEM. Grid samples were loaded onto a standard low‐background TEM double tilt holder (Thermo Fisher Scientific, Waltham, MA USA). TEM images were acquired using a Titan 80–300 keV and Tecnai G2 80–200 keV TEMs (Thermo Fisher Scientific, Waltham, MA USA; formerly produced by FEI) equipped with a 4096 × 4096 pixels^2^ OneView CMOS camera (Gatan, Inc., Pleasanton, CA, USA). TEM images were processed using Digital Micrograph (DM) (Gatan, Inc., Pleasanton, CA, USA) and open‐source ImageJ (National Institutes of Health) software to enhance the contrast and brightness.

### Thermoelectric Measurements

Seebeck coefficient of TE hybrid films was measured with a Portable Thermoelectric Meter (PTM‐3, Joule Yacht) with 0.1 µV K^−1^ resolution of Seebeck coefficient, and temperature gradient (∆T) of 25 ⁰C between hot and cold probe ends (≈5 mm apart). The company claims a measurement error of ±7%. To ensure the consistency of the results, the thermoelectric properties were verified using a cryostat probe station, as depicted in Figure S12 (Supporting Information). The thicknesses of fabricated films were estimated by Alpha‐Step IQ surface profiler. Films were scratched at the center with a toothpick and scanned at various points to analyze the uniform thickness of the films. Electrical conductivity measurements were carried out with a typical four‐point probe set up using Keithley 2450 source meter. After obtaining the IV characteristics curves, sheet resistance was multiplied with geometric factor as reported in the literature,^[^ ^35^ ^]^ which then were multiplied with thickness of films to obtain resistivity (*ρ*) values, then electrical conductivity (σ) of films was calculated by using equation 𝜎 = 1/𝜌. Finally, the power factor (PF) of TE materials was calculated as *PF* = *S* ^2^𝜎. Several samples were used for the measurements, and each sample was tested at least 4 times from different points to ensure the reproducibility of the results.

## Conflict of Interest

The authors declare no conflict of interest.

## Supporting information

Supporting Information

## Data Availability

The data that support the findings of this study are available from the corresponding author upon reasonable request.
