# Supplementary material for: Oxidation Control to Augment Interfacial Charge Transport in Te‐P3HT Hybrid Materials for High Thermoelectric Performance
Source: Adv Sci (Weinh). 2024 Jul 23;11(35):2400802. doi: 10.1002/advs.202400802 (PMC11425214; doi:10.1002/advs.202400802)
Supplement: Supplementary file 1 — Supporting Information [file ADVS-11-2400802-s001.docx]

# Supporting Information

Oxidation Control to augment Interfacial Charge Transport in Te-P3HT Hybrid Materials for High Thermoelectric Performance

Syed Zulfiqar Hussain Shah^1,2*^, Ding Zhenyu^5^, Zainul Aabdin^1^, Weng Weei Tjiu^1^, Jose Recatala-Gomez^3^, Haiwen Dai^3^, Yang Xiaoping^5^**,** Repaka Durga Venkata Maheswar^1^, Wu Gang^4#^, Kedar Hippalgaonkar^1, 3#^, Iris Nandhakumar^2#^, Pawan Kumar^1#^,

1. Institute of Materials Research and Engineering, Agency for Science Technology and Research

(A*STAR), Singapore 138634, Republic of Singapore.

2. Department of Chemistry, University of Southampton, Southampton SO17 1BJ, United Kingdom.

3. School of Materials Science and Engineering, Nanyang Technological University, 50 Nanyang

Avenue, Block N4.1, 639798, Singapore.

4. Institute of High-Performance Computing, Agency for Science Technology and Research

(A*STAR), Singapore 138632, Republic of Singapore.

5. High Magnetic Field Laboratory, Chinese Academy of Sciences, University of Science and Technology of China, No.96, JinZhai Road Baohe District, Hefei, Anhui, 230026, P.R. China

* First Author, Corresponding Authors^#^


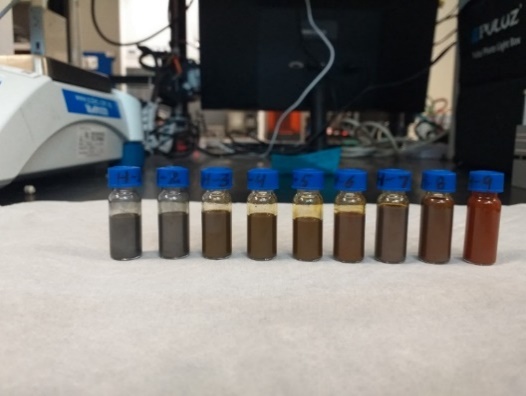

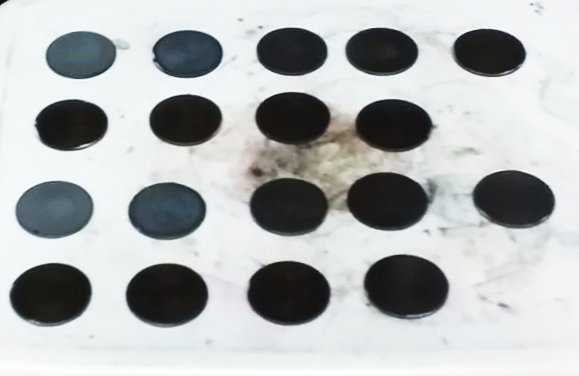


Fig. S1. Dispersion of TeNWs in chloroform with varying weight percent of TeNWs (10 wt% to 90 wt%) in P3HT matrix (top-image) and fabricated films of P3HT-TeNWs hybrid nanocomposites (bottom image). Films were fabricated via drop casting on quartz substrates in nitrogen filled glovebox environment with O_2_ <1ppm, and H_2_O <1ppm. The optical bottom image depicts two batches of hybrid films, (the 1^st^  & 2^nd^ row belongs to first batch while the 3^rd^ & 4^th^ row of second batch), where the highest concentration of nanowires begins from the top-left corner and gradually diminishes towards the bottom-right corner.


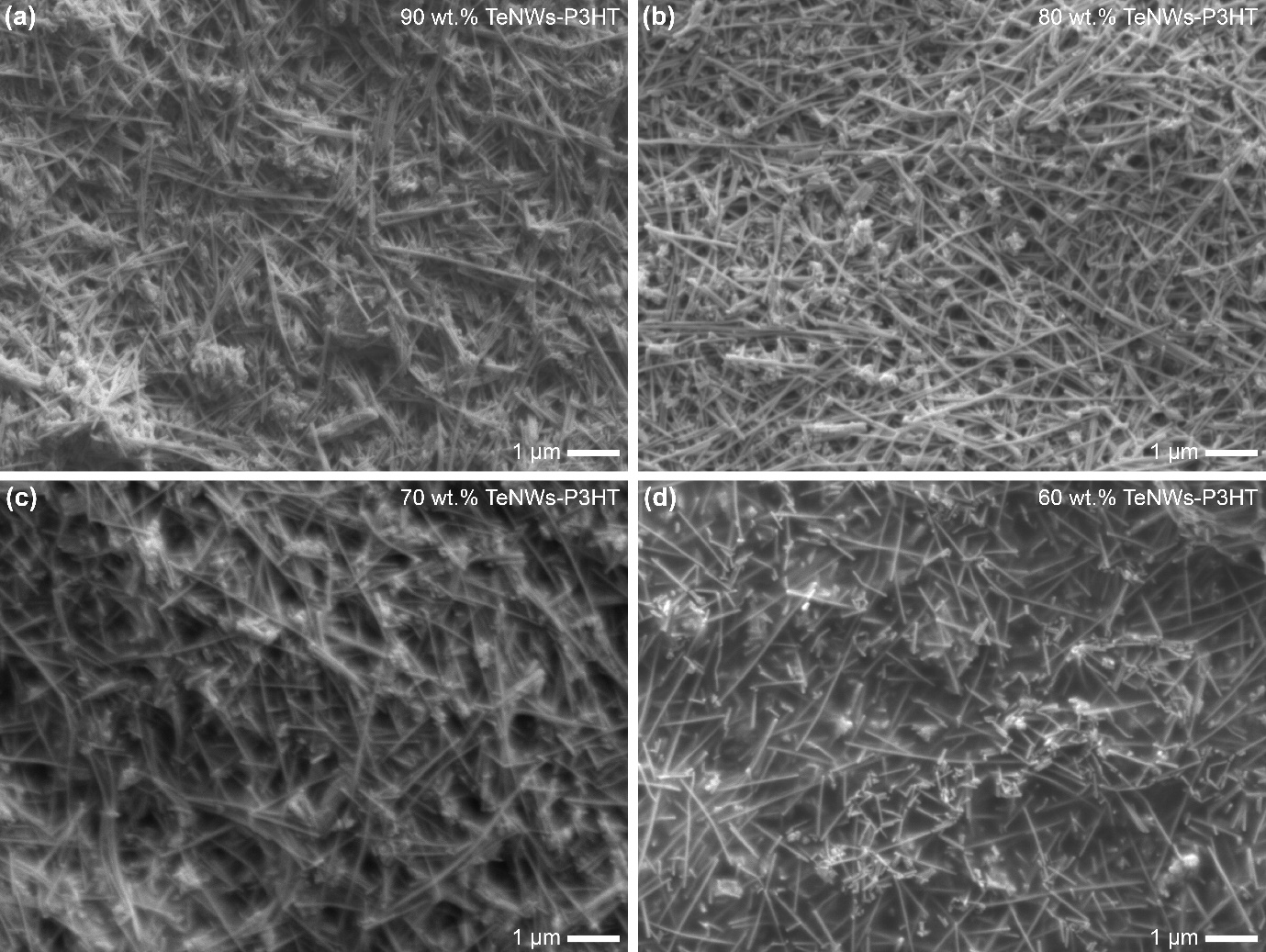


Fig. S2: SEM images of P3HT-TeNWs nanocomposite hybrid films using various TeNWs concentration in P3HT matrix (a) 90 wt.%, (b) 80 wt.%, (c) 70 wt.%, and (d) 60 wt.% exhibiting a homogenous dispersion of TeNWs within the P3HT matrix.


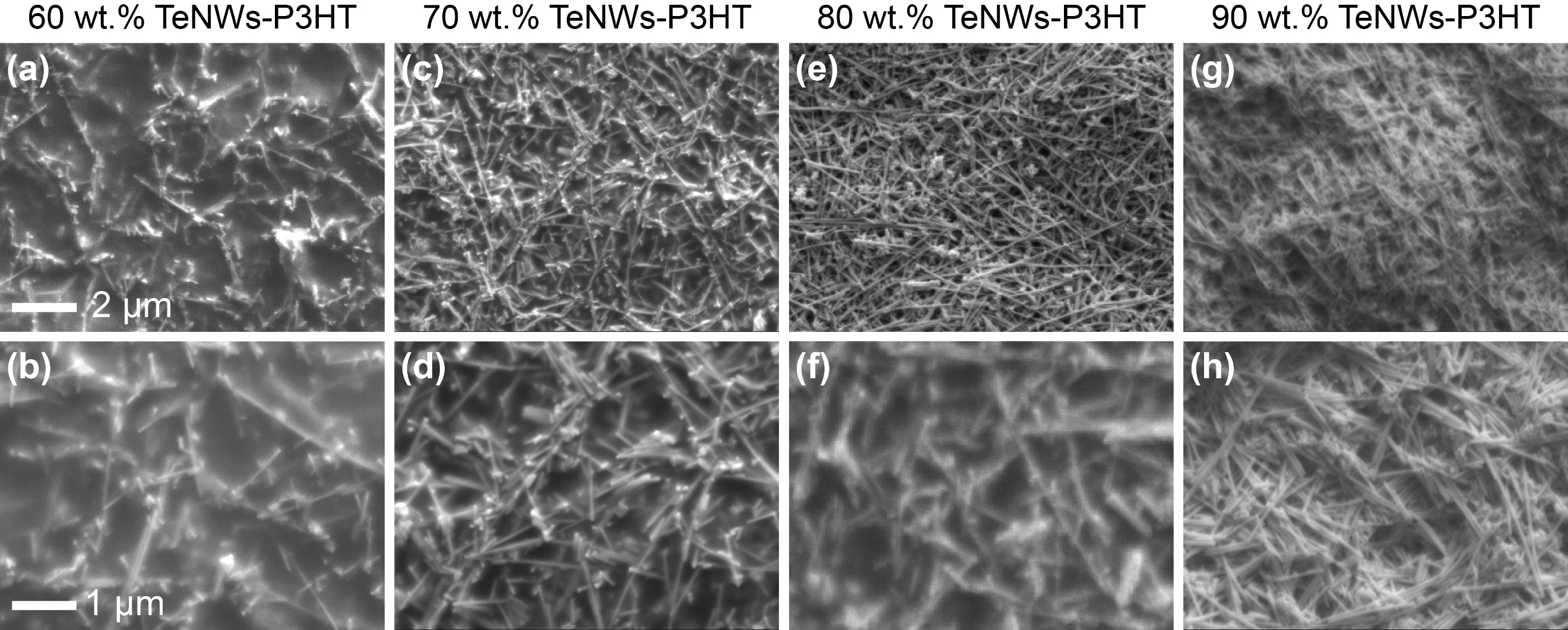


Fig. S2-b: SEM images of P3HT-TeNWs nanocomposite hybrid films using varying concentration of TeNWs in P3HT matrix (a,b) 60 wt.%, (c,d) 70 wt.%, (e,f) 80 wt.%, and (g,h) 90 wt.%. Scale bars: (a,c,e,g) 2µm, (b,d,f,h) 1µm. The images demonstrate a homogenous dispersion of TeNWs within the P3HT matrix.


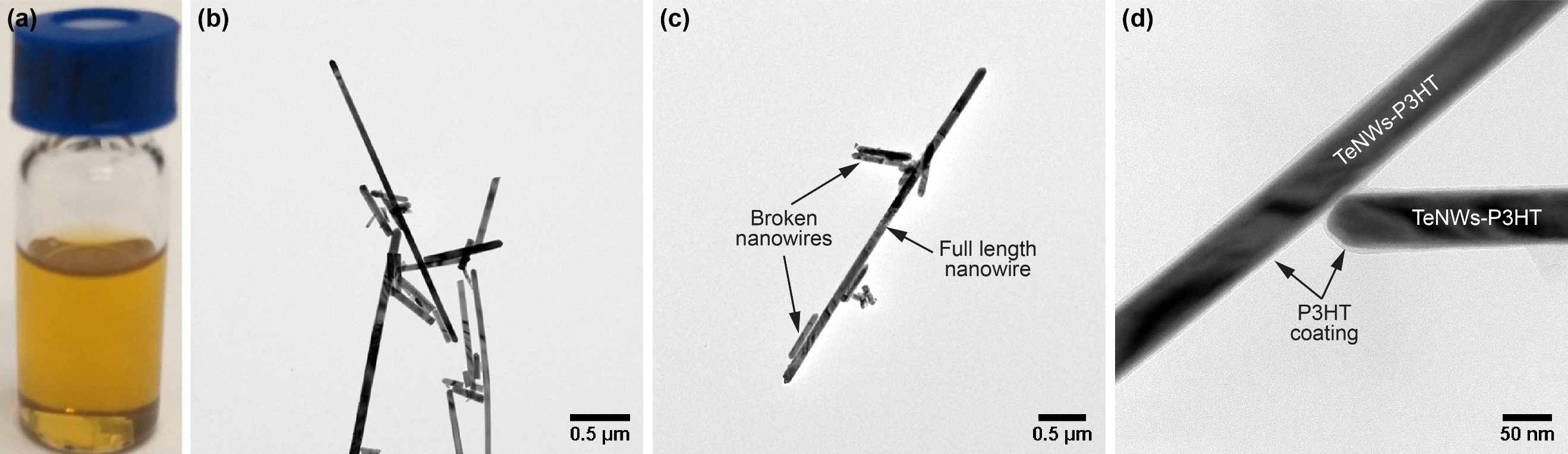


Fig. S3. (a) Dilute dispersion solution of P3HT-TeNWs (50 wt.% TeNWs in P3HT matrix) nanocomposite in chloroform used to prepared grid samples and perform TEM characterization. (b), (c) and (d) Low-magnification TEM images of P3HT-TeNWs hybrid nanocomposites showing a few nanowires. The amorphous layer on the surface of nanowires in (d) indicates the conformal coating of polymer on nanowires.


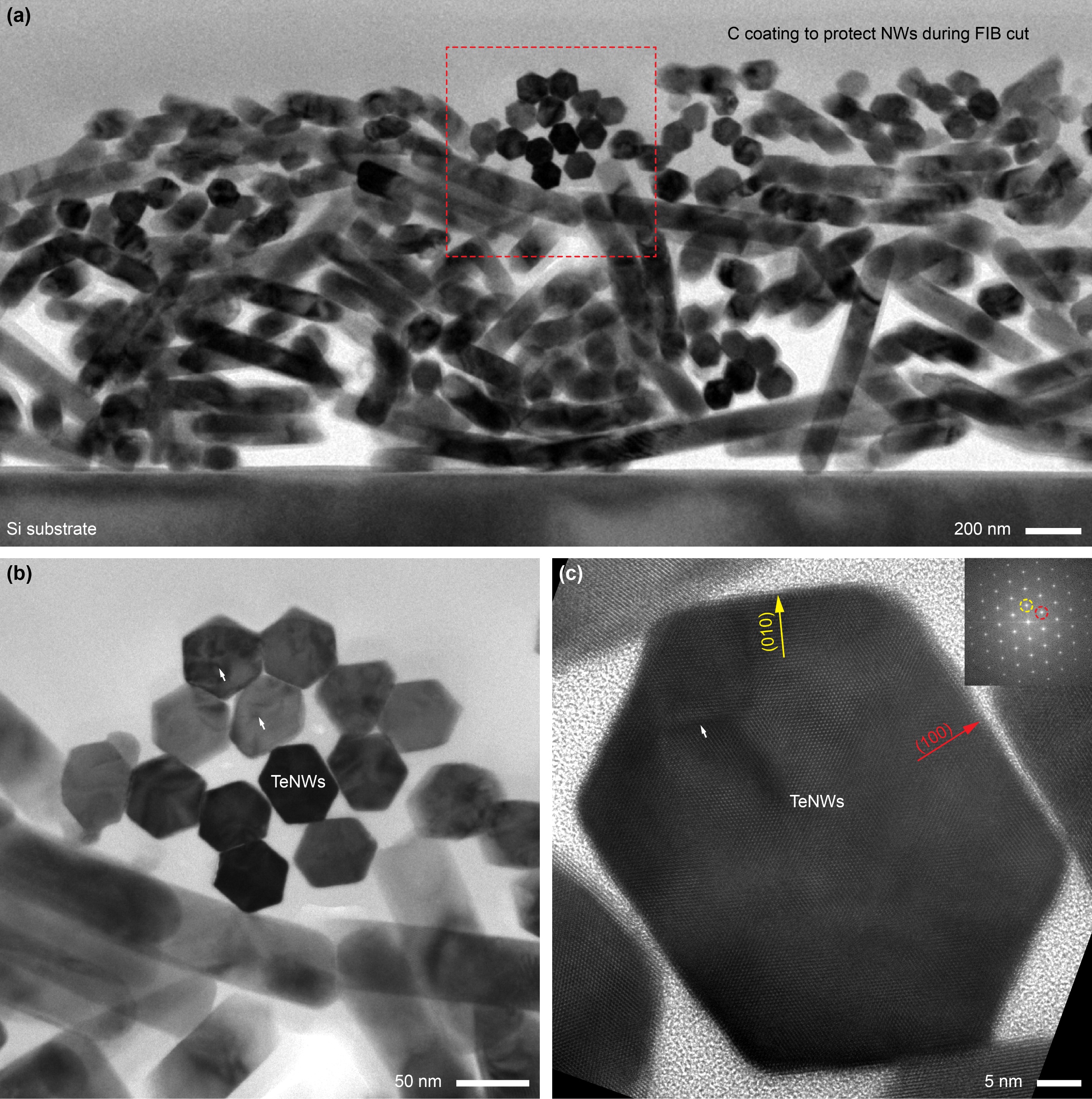


Fig. S4. (a-b) Low-magnification TEM images of TeNWs showing random distribution of the NWs on a Si substrate and morphology of individual NWs. (c) High-resolution cross-section TEM image of a single TeNW showing the hexagonal shape of NW bounded by the six (100) facets confirmed by the lattice fringes and corresponding FFT (inset in (c)). The core of the NWs is single crystalline with little amount of defect or dislocation (*white arrows in* (b) *and* (c)). NWs were dispersed in ethanol and drop casted on a Si substrate followed by C-coating and then a cross-section sample was prepared via FIB cut.


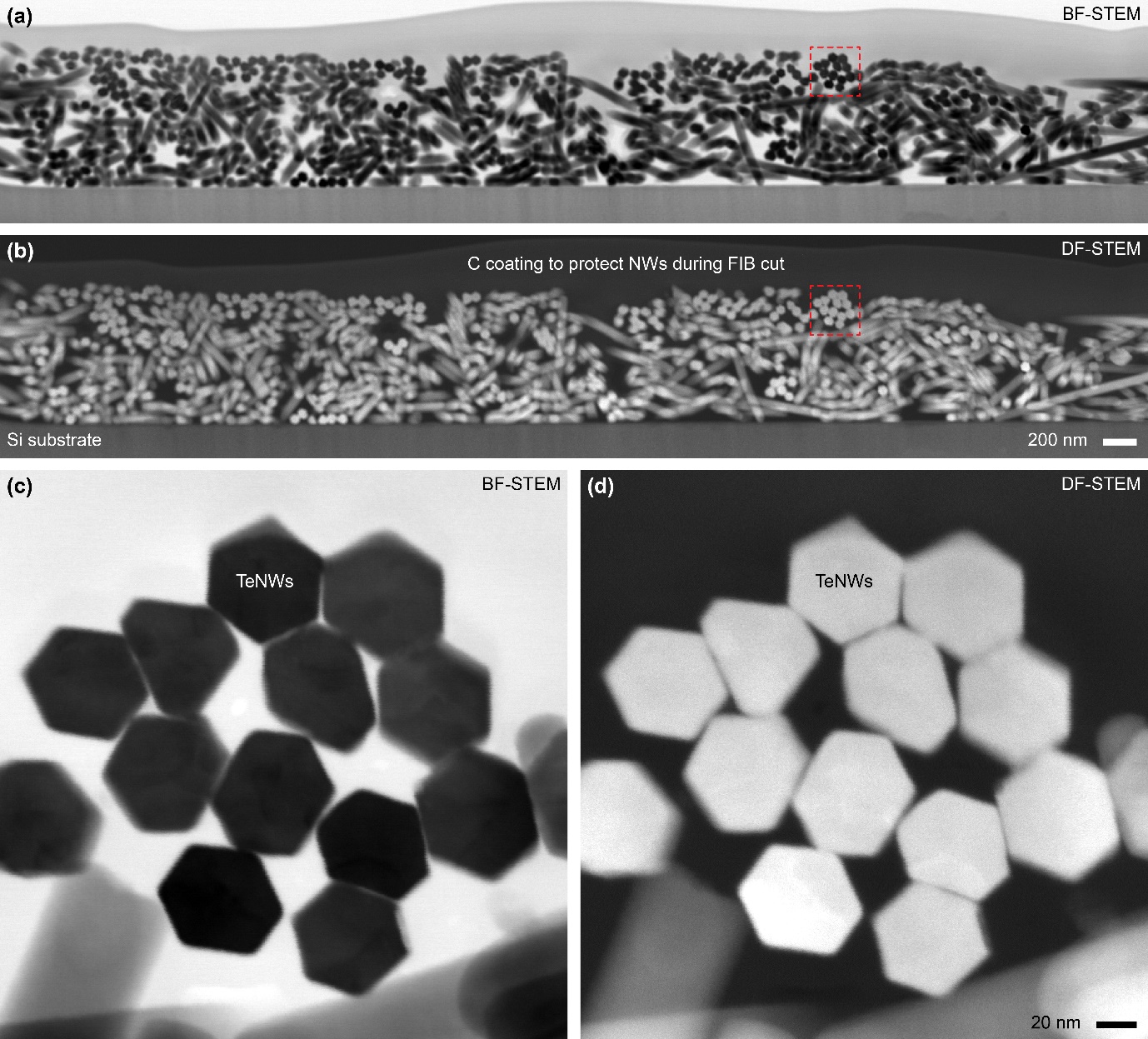


Fig. S5. (a) Bright-field (BF) and (b) dark-field (DF) STEM images of TeNWs at low-magnification showing random distribution and texture of the NWs. High-magnification (c) BF-STEM and (d) DF-STEM images of TeNWs from selected areas (*red dotted squares*) showing a perfect cross-sectional view of the NWs. Most of the NWs when seen in cross-section are in perfect hexagonal shape with flat facets or edges and free from any contamination or oxidation.


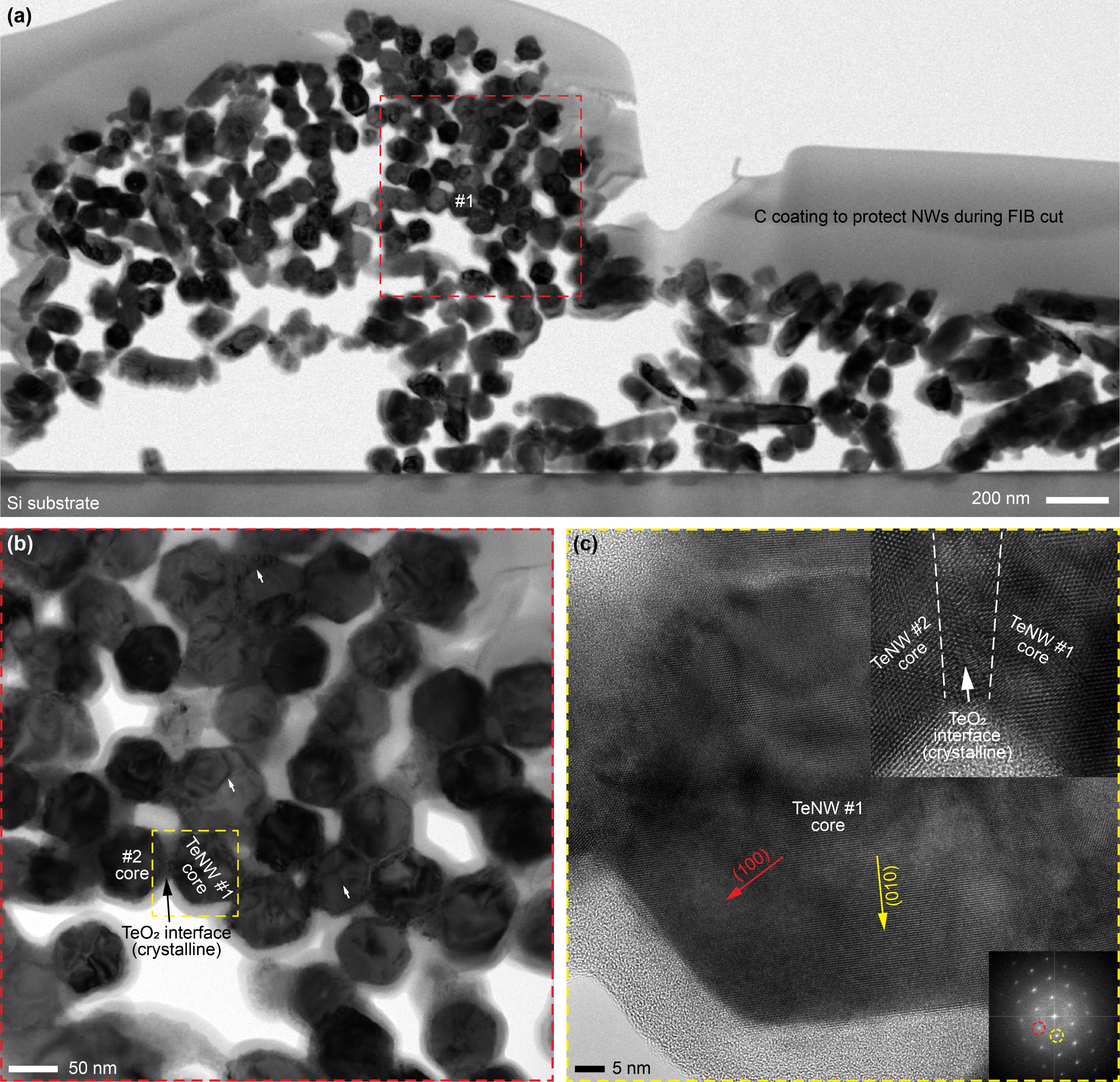


Fig. S6. (a-b) Low-magnification TEM images of TeO_2_-NWs showing the random distribution of TeNWs on a Si substrate and morphology of individual NWs. A comparison of the TEM images of TeNWs [Fig. S4 (b)] and TeO_2_-NWs [Fig. S6 (b)] shows that the oxide formation introduces more defects in the core of nanowires (*white arrows in* (b)). (c) High-resolution cross-section TEM image of TeNWs core and the interface layer between the two NWs. The interface layer is crystalline and appears to be TeO_2_ due to surface oxidation. Surprisingly, the oxide that are not in close proximity of the NWs surface appears to be amorphous, however, the interface oxide appears to be polycrystalline.


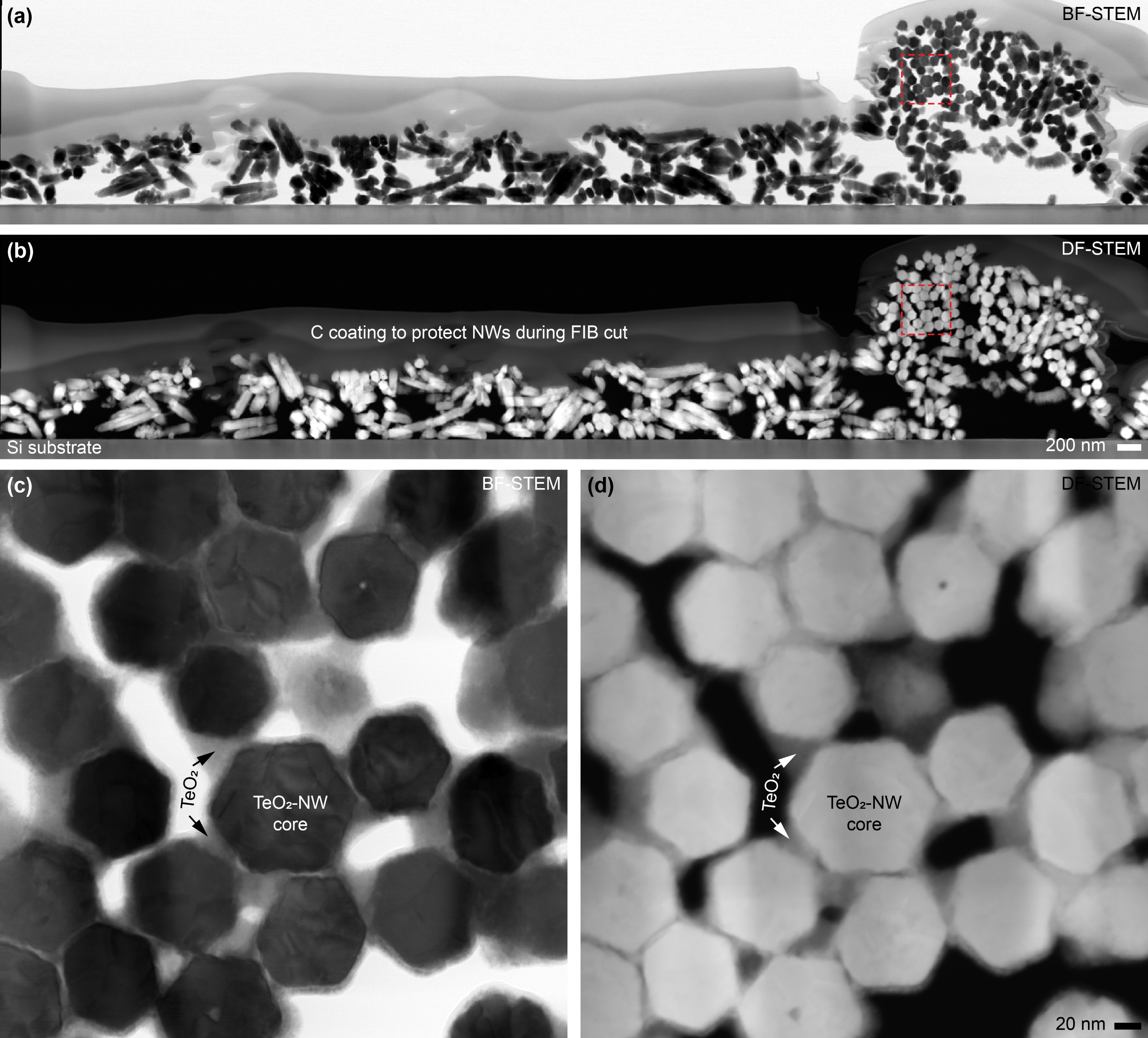


Fig. S7. (a) Bright-field (BF) and (b) dark-field (DF) STEM images of TeO_2_-NWs at low-magnification showing random distribution and texture of the NWs. High-magnification (c) BF-STEM and (d) DF-STEM images of TeO_2_-NWs from selected areas (*red dotted squares*) showing a perfect cross-sectional view of the NWs. Most of the NWs when seen in cross-section are in perfect hexagonal shape with flat facets or edges. However, in contrast to TeNWs, in this case we clearly see surface roughness due to surface oxidation.


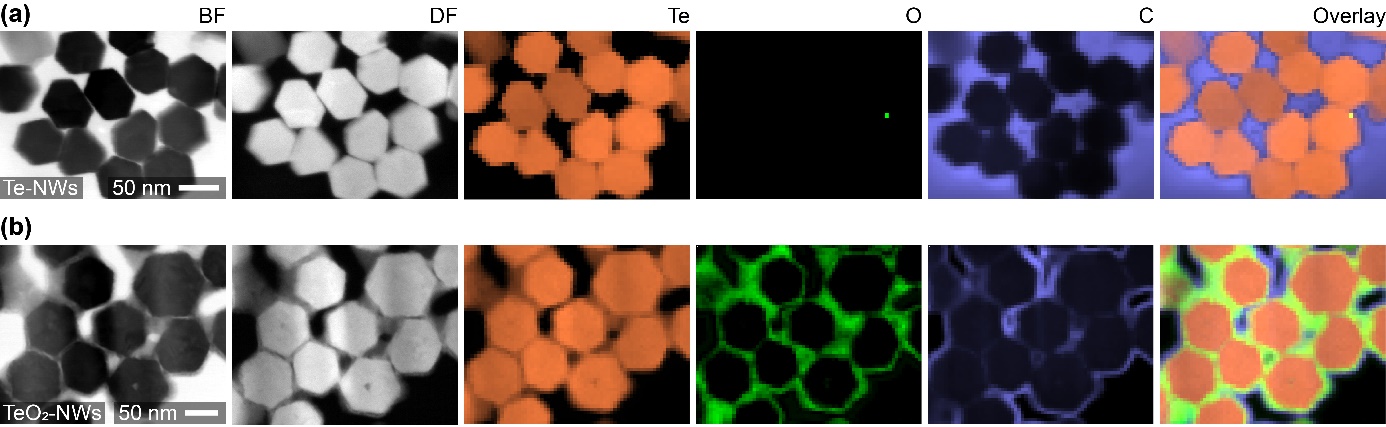


Fig. S8. BF-STEM, DF_STEM images, corresponding elemental (Te, O, C) EELS maps, and overlay (Te + O + C) maps (a) Te-NWs and (b) TeO_2_-NWs. This figure is like Fig. 3; however, this is in low magnification showing a larger area. Here we clearly see that there is no oxide formation for the former case. In the latter case we clearly see oxide formation and the oxide thickness is not uniform across a given NW, it varies and most of the oxide exists in between the NWs i.e., as an interface layer between NWs.

Fig. S9. X-ray diffraction (XRD) patterns of TeO_2_-NWs, and TeNWs films.

Fig. S9 shows the XRD patterns of TeNWs, TeO_2_-NWs films. A comparison with the simulated XRD pattern (ICDD PDF Card 00-036-1452) for Te indicates that the synthesized TeNWs correspond to pure phase Tellurium with Hexagonal crystal system and no other impurities being present.^[1]^

Fig. S9-b: High resolution X-ray photoelectron spectroscopy (XPS) spectra of O 1s of tellurium nanowires. The dots indicate raw data, solid black curve shows envelope and a green line background.


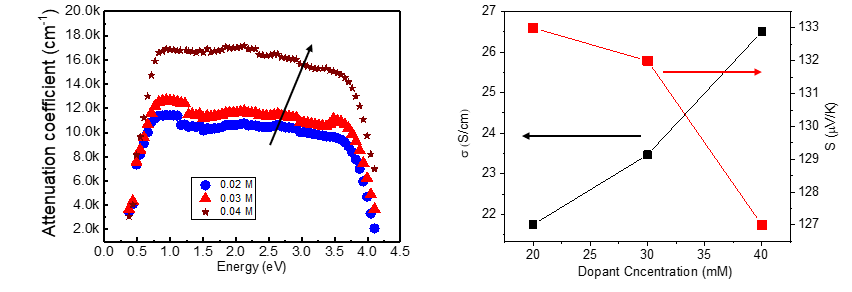


Fig. S10: UV-Vis and TE properties of P3HT-TeNWs hybrid films with varying dopant concentrations.

We have shown in Fig. 4(c), Seebeck is dropping with increasing dopant concentration because Seebeck in inversely proportional to carrier concentration (S α $\frac{1}{n^{2/3}}$).^[17]^ Apart from this, we tried UV-Vis (Fig. S10), where it has been shown an enhancement in attenuation coefficient with increasing molar content, which is another qualitative proof of increasing carrier concentration in the system.


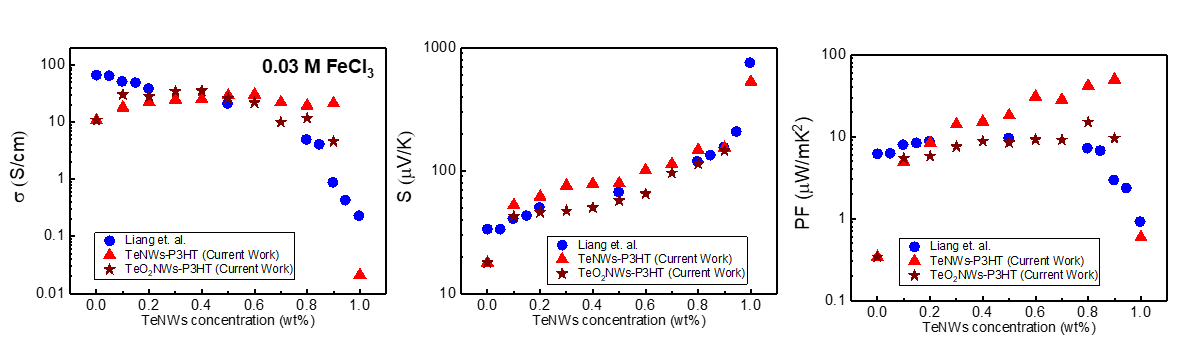


Fig. S11: **Comparison of** TE properties of 30% FeCl_3_ doped P3HT-TeNWs hybrid films with literature.


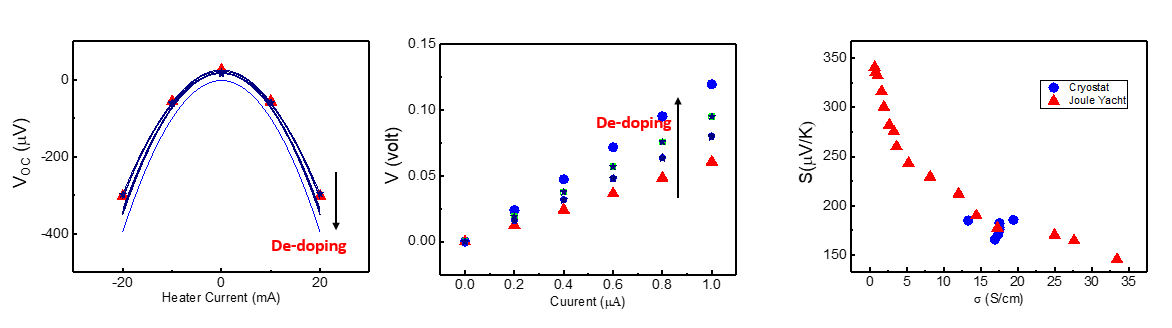


Fig. S12: A comparison of TE properties measured via cryostat probe station and portable Seebeck Tester (PMT) on de-doping the 0.06M FeCl_3_ hybrid film containing 90% Te and 10% P3HT.

In this study, we conducted thermoelectric measurements in ambient conditions utilizing a Seebeck coefficient Tester (PTM-3) manufactured by Wuhan Joule Yatch Technology, a device commonly employed by other researchers. ^[18,19]^ To ensure result consistency, we performed de-doping on hybrid films using a cryostat probe station. This allowed us to compare the results obtained with those from the PTM-3, as illustrated in Figure S12.

| *Sample* | *σ (S/cm)* | *S (µV/K)* | *PF (µW/mK^2^)* |
| --- | --- | --- | --- |
| TeNWs (100 wt%) | 0.021 | 528.8 | 0.59 |
| TeNWs (90 wt%) | 0.11 | 460.50 | 2.26 |
| TeNWs (80 wt%) | 0.089 | 456.95 | 1.87 |
| TeNWs (70 wt%) | 0.022 | 438.28 | 0.42 |
| TeNWs (0 wt%) | 10.725 | 17.80 | 0.34 |

Table S1. Summary of the TE properties of undoped TeNWs-P3HT nanocomposites hybrid films, pure TeNWs film and 0.03M FeCl_3_ doped P3HT film (0 wt% TeNWs).

As indicated in Table S1, oxidation control in thin films of TeNWs and TeNWs-P3HT composite led to the observation of a conductive nature without the need for doping, resulting a power factor of 2.26 µW/mK^2^ for the hybrid sample with a TeNWs concentration of 90% (by weight) in the P3HT matrix.

Binary model with series and parallel combinations was used to explained Seebeck and electrical conductivity trend. According to this model:

## Molecular dynamics (MD) Simulations

To begin our study, we adopted the experimental lattice constants for Te and TeO_2_ and chose specific surface orientations. The (10-10) plane was selected for Te due to its lowest surface energy, while the (110) plane of tetragonal *α*-TeO_2_ ^[1]^ was chosen for TeO_2_ based on the experimental findings.^[2,3]^ The lattice parameters for Te are *a* = 4.19 Å, *b* = 5.98 Å, *c* in the vacuum direction; *α* = *β* = *γ* = 90°. The lattice parameters for TeO_2_ are a = b = 4.83 Å, c = 7.65 Å; α = β = γ = 90°.

To examine the morphology and configuration of P3HT on Te and TeO_2_ nanowire surfaces, we constructed supercells of the respective surfaces. An 18×15 supercell (8.0×8.9 nm^2^) comprising 2430 Te atoms was created for the Te (10-10) surface. Similarly, an 11×12 supercell (8.4×8.2 nm^2^) containing 5412 atoms (Te_1980_O_3432_) was constructed for the TeO_1.73_ (110) surface. The surface thickness was determined to be 9.77 Å for Te and 10.66 Å for TeO_1.73_. P3HT was represented by 16 molecular chains of (3HT)_12_ oligomers.

Simulations were performed using the LAMMPS simulation package.^[4]^ The Nosé-Hoover thermostat and barostat were employed to control the system's temperature and pressure. The interatomic forces were evaluated using the Condensed-phase Optimized Molecular Potentials for Atomistic Simulation Studies (COMPASS) potential.^[5–7]^ Electrostatic forces were calculated using the Ewald summation method,^[8]^ and van der Waals forces were treated using an atom-based approach with a 12 Å atomic cutoff distance for van der Waals interactions.

During the equilibration process, different timesteps were used for Te/P3HT and TeO_2_/P3HT systems. The Te/P3HT system employed a timestep of 0.8 fs, while the TeO_2_/P3HT system used a timestep of 2.0 fs. The equilibration process involved heating the systems from 250 K to 450 K over 2,500,000 steps, followed by a maintenance period of 10,000,000 steps at 450 K and subsequent cooling from 450 K to 280 K over 2,500,000 steps. In the production stage, the systems were maintained at 280 K for 20 ns for Te/P3HT and 10 ns for TeO_2_/P3HT, using a timestep of 1.0 fs.

## DFT Calculations

Based on the results obtained from molecular dynamics calculations, we constructed the Te/P3HT structure and three different TeO_2_/P3HT structures for density functional theory (DFT) calculations. The lattice constants used in the DFT simulations were consistent with those employed in the molecular dynamics (MD) simulations. To model the Te (10-10) and TeO_2_ (110) substrates, we created slabs with thicknesses of 9.77 Å and 10.66 Å, respectively, ensuring a 25 Å vacuum slab to eliminate interlayer interactions. The dipole correction was introduced to compensate the potential dipole field of non-symmetric slabs by an electrostatic potential step in the vacuum region.

For the Te/P3HT structure, we constructed a $\sqrt{2}\times\sqrt{2}$ supercell of the Te (10-10) surface with two (3HT) units in each supercell. This configuration corresponds to the most probable distribution and orientation of the P3HT backbones, as observed in the MD simulation, with an angle of 50° (-40°) relative to the Y-axis.

Regarding TeO_2_/P3HT, we designed a 1×3 supercell of the TeO_2_ (110) surface with two (3HT) units per cell. This supercell aligns the P3HT backbone along the X-axis direction, as observed in the MD simulation. Additionally, a $2\sqrt{2}\times3\sqrt{2}$ supercell of the TeO_2_ (110) surface was created, accommodating eight (3HT) units. In this configuration, the P3HT backbones are oriented at an angle of either -40° or 50° relative to the Y-axis. Finally, a 3×6 supercell of the TeO_2_ (110) surface with twelve (3HT) units was established to align the P3HT backbones along the Y-axis.

The band structures and electron density differences between the surfaces and P3HT were computed using Density Functional Theory (DFT) within the all-electron FHI-aims package.^[9]^ The Perdew-Burke-Ernzerhof (PBE) exchange-correlation function^[10]^ was utilized throughout the DFT calculations. Long-range van der Waals interactions were taken into consideration via the Tkatchenko and Scheffler (TS) scheme.^[11]^ The scalar-relativistic effect was treated at the level of atomic ZORA approximation.^[9]^ Monkhorst-Pack sampling was employed for the *k*-point sampling with a spacing of 0.02 Å^-1^. The atomic internal coordinates were optimized using the conjugate gradient technique until the maximum residual force component on each atom did not exceed 0.005 eV/Å in the final structures.

In order to study the charge transfer and de-doping effect, the Bader charges^[12]^ were calculated using the Bader charge analysis program developed by Henkelman group.^[13, 14]^ The electron density differences were visualized using Visualization for Electronic and Structural Analysis (VESTA).^[15]^

## MD results for P3HT/Te

Figure S13 illustrates the initial and final states of the Te/P3HT system, along with the concentration distribution of all atoms in P3HT along the Z-direction. Initially, the P3HT configuration is spatially disordered, spanning a range of 50-80 Å. However, in the final state, two distinct peaks are observed near the Te surface, indicating a localized concentration of P3HT in close proximity to Te. Additionally, the Z-direction heights of the carbon (C) atoms on the 16 (3HT)_12_ chains are depicted in the left panel of Figure S15. A clear layered distribution in the Z-direction is evident, with variations attributed to uneven interlayer stacking. The majority of the chains are concentrated within the first and second layers, consistent with the two peaks observed in the right panel of Figure 1.

During the production stage in MD simulations, the 16 (3HT)_12_ chains exhibit a linear alignment in the XY plane. This is demonstrated by calculating the angles between the backbones and the Y-axis in the last 1 ns, as shown in the left panel of Figure S16. The analysis, based on the coordinates of the S atoms on the mainchains and employing linear regression processing, reveals that the chains are primarily distributed around -40° and 50°, which are identified as the preferential orientations for the P3HT backbones on the Te surface. This templating effect can be further visualized from the snapshots depicted in Figure S17 (a) and (b).

Additionally, the inter-chain distances in Figure S17 (a) were calculated by the distance between the parallel backbones of the adjacent P3HT chains. The resulting distances for d_1_, d_2_, d_3_, d_4_, and d_5_ are 18.5 Å, 16.8 Å, 18.5 Å, 17.3 Å, and 19.1 Å, respectively, with an average distance of approximately 18 Å. Therefore, a Te/P3HT structure was constructed for further DFT calculations, enabling the analysis of the charge distribution, as depicted in Figure S17 (c) and (d).

## MD results for P3HT/TeO_2_

Figure S14 demonstrates that the P3HT/TeO_2_ system exhibits similar characteristics to the P3HT/Te system, with both showing distinct templating effect on the inorganic surfaces. However, the right panel of Figure 4 reveals that the orientation of P3HT on the TeO_2_ surface is more diverse compared to that on the Te surface, which only exhibits essentially a single type of orientation. This diversity in P3HT orientation leads to more uneven layer stacking, as shown in the right panel of Figure 3.

In the right panel of Figure S16, we observe distinct distributions near 0°, 50°, -40°, 75°, and -75°, indicating the presence of several preferential orientations. Accordingly, we constructed four DFT structures (Figure S18 (c), (d), (e), and (f)) to represent these orientations: Y-axis direction (0°), X-axis direction (90°), -40° and 50° relative to the Y-axis, respectively. We observed that constructing a small commensurable model to accommodate both P3HT and TeO_2_ with minimal strain poses a challenge. Consequently, the largest interfacial model (-40°) contains 1161 atoms. In contrast, when the backbone of the P3HT aligns with the X-axis (90° model), the number of atoms decreases to 173. The considerably larger interfacial model for the TeO_2_ substrate indicates that the potential energy surface (PES) of P3HT on TeO_2_ is significantly more complex than that on Te. Even minor thermal perturbations can lead to the rotation of the P3HT chains within this intricate environment. Remarkably, these findings align with the observations from our MD simulations.

The distances between the mainchains were calculated from the distances highlighted in Figure S18 (a). Specifically, d'_1_ represents the distance of 19.2 Å between the backbones at an orientation angle of 50°. Furthermore, d'_2_, d'_3_, and d'_4_ correspond to the distances of approximately 16 Å, 17.9 Å, and 20.7 Å, respectively, between the backbones that are nearly parallel to the Y-axis. The average distance between these chains is approximately 18.2 Å.

## Charge transfer and de-doping effect

The analysis of charge density difference (see Figure S19 (a) and (b)) uncovers a minor charge transfer from the Te (10-10) surface to the backbone of P3HT. This transfer is further quantified through Bader charge analysis, revealing that each 3HT molecule gains 0.028 $e_{0}$ from Te.

On the other hand, when P3HT is in contact with TeO_2_, the charge transfer becomes more pronounced across the four orientations (see Figure S19 (c) and (d)). For each of the four preferential orientations (0°, 90°, -40°, and 50° relative to the Y-axis), Bader charge analysis reveals similar charge transfers of 0.095 $e_{0}$, 0.100 $e_{0}$, 0.078 $e_{0}$, and 0.138 $e_{0}$, respectively. We suspect that the dipole effect of the TeO_2_ substrate plays a significant role in promoting these charge transfer processes. The presence of a dipole moment in the TeO_2_ surface may induce localized electric fields, thus facilitating the efficient transfer of charges between the inorganic substrate and the P3HT polymer, leading to the observed large charge transfer in the case of TeO_2_ substrates.

Using the volume of polymorph I CA-100 as reported in Ref. 16, we determined the number of electrons transferred per unit volume of P3HT when a single layer covers the inorganic surface, as shown in Table S2.

Due to the similarity of the charge transfer in all the interfacial models, we will study the electronic band structures in the P3HT/TeO_2_ (90°) model in details. The band structures in Figure 8 also support these findings, showing that the band contributed by TeO_2_ is slightly above the Fermi level around A-Y special k-point line, while the band contributed by P3HT is just below the Fermi level around Γ point. This finding confirms the occurrence of charge transfer between P3HT and TeO_2_.

Additionally, we conducted calculations of the highest occupied molecular orbital (HOMO) and lowest unoccupied molecular orbital (LUMO) orbitals in Figure S19. The distribution of the orbitals demonstrates the involved orbitals during charge transfer. Clearly, the distribution of the HOMO orbital coincides with the charge density difference in Figure S19 (c)&(d) and confirms that the charge transfer occurs between the TeO_2_ surface and the backbone of P3HT.

| Systems | Charge transfer per (3HT) unit ($\boldsymbol{e}_{\boldsymbol{0}}$) | De-doping effect ^a^ ($\boldsymbol{\times}\boldsymbol{10}^{\boldsymbol{20}} \boldsymbol{e}_{\boldsymbol{0}}$/cm^3^) |
| --- | --- | --- |
| P3HT on Te | 0.028 | 1.14 |
| P3HT on TeO_2_ (90◦) | 0.100 | 4.06 |
| P3HT on TeO_2_ (0◦) | 0.095 | 3.86 |
| P3HT on TeO_2_ (50◦) | 0.138 | 5.59 |
| P3HT on TeO_2_ (-40◦) | 0.078 | 3.17 |

Table S2. Interfacial charge transfer calculations from DFT and Bader charge analysis (^a^ volume of each (3TH) unit: 2.46$\times{10}^{-22}$ cm^3^)


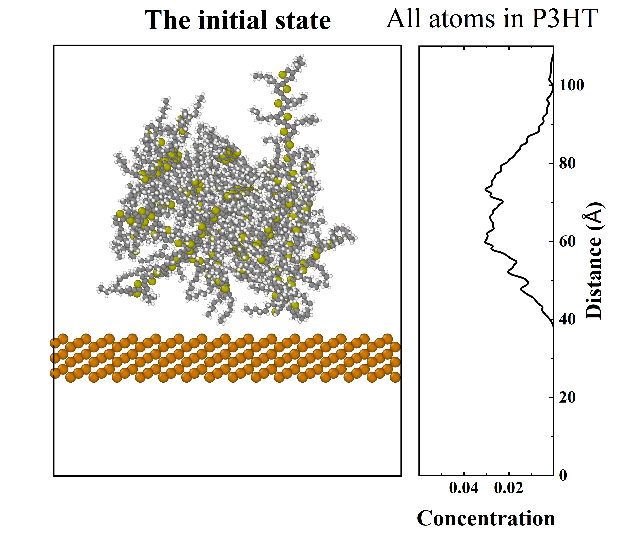

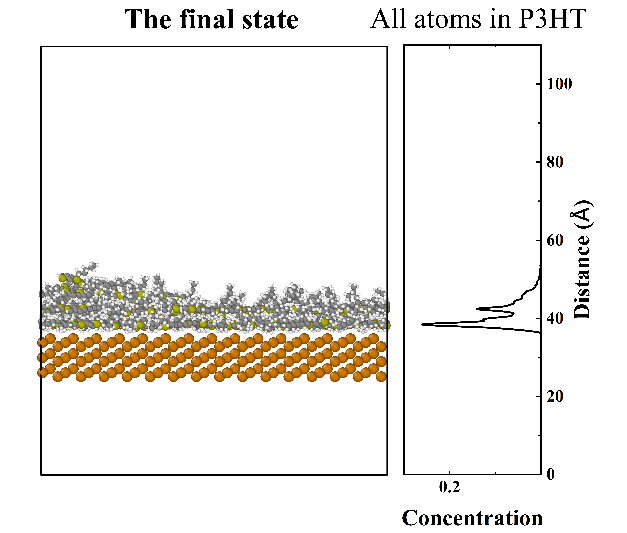


Fig. S13. Initial (left) and final (right) structures for MD simulation and corresponding concentration profiles of P3HT/Te.


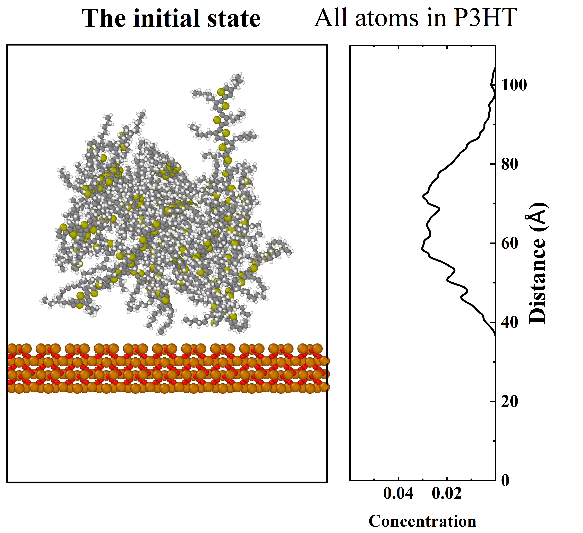

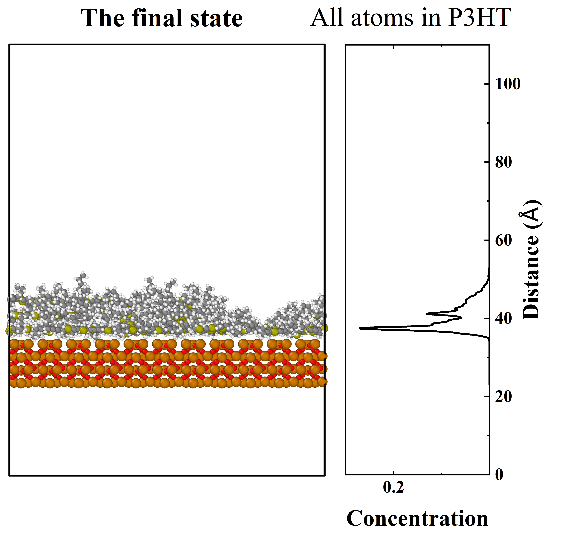


Fig. S14. Initial (left) and final (right) structures for MD simulation and corresponding concentration profiles of P3HT/TeO_2_.


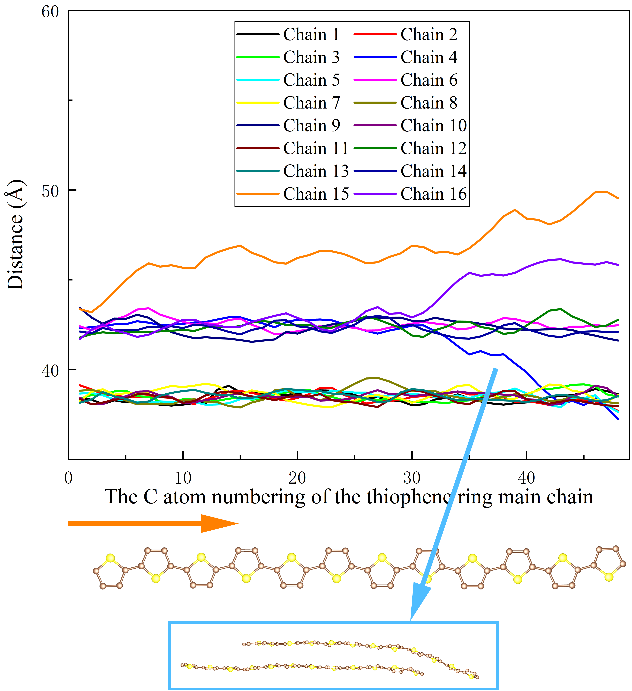

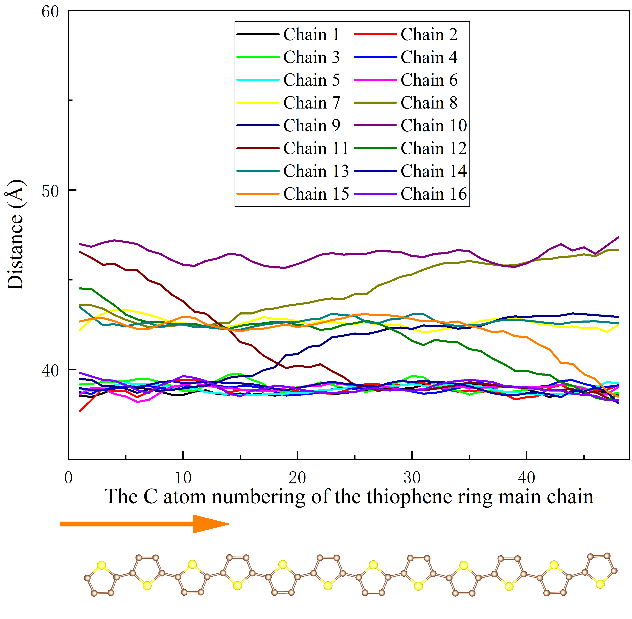


Fig. S15. The Z-direction heights of the C atoms on the backbones of the 16 (3HT)_12_ oligomers in P3HT/Te (left) and P3HT/TeO_2_ (right); the blue box illustrates the height difference in the Z-direction caused by the uneven stacking method.


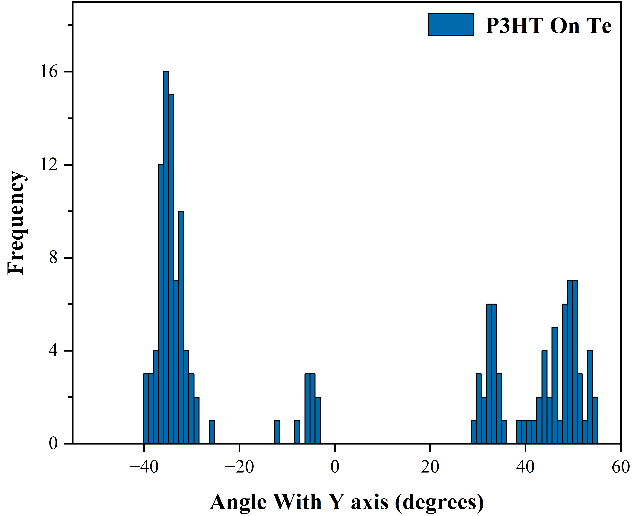

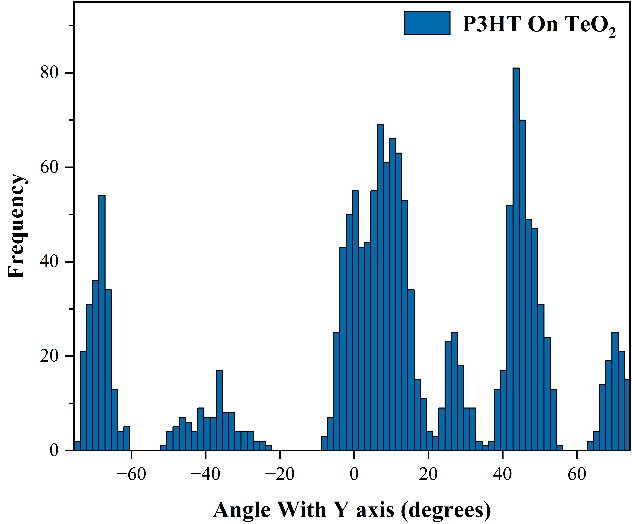


Fig. S16. The statistical distribution of the angles between the backbones of the 16 (3HT)_12_ oligomers and the Y-axis in the XY plane in the last 1 ns.


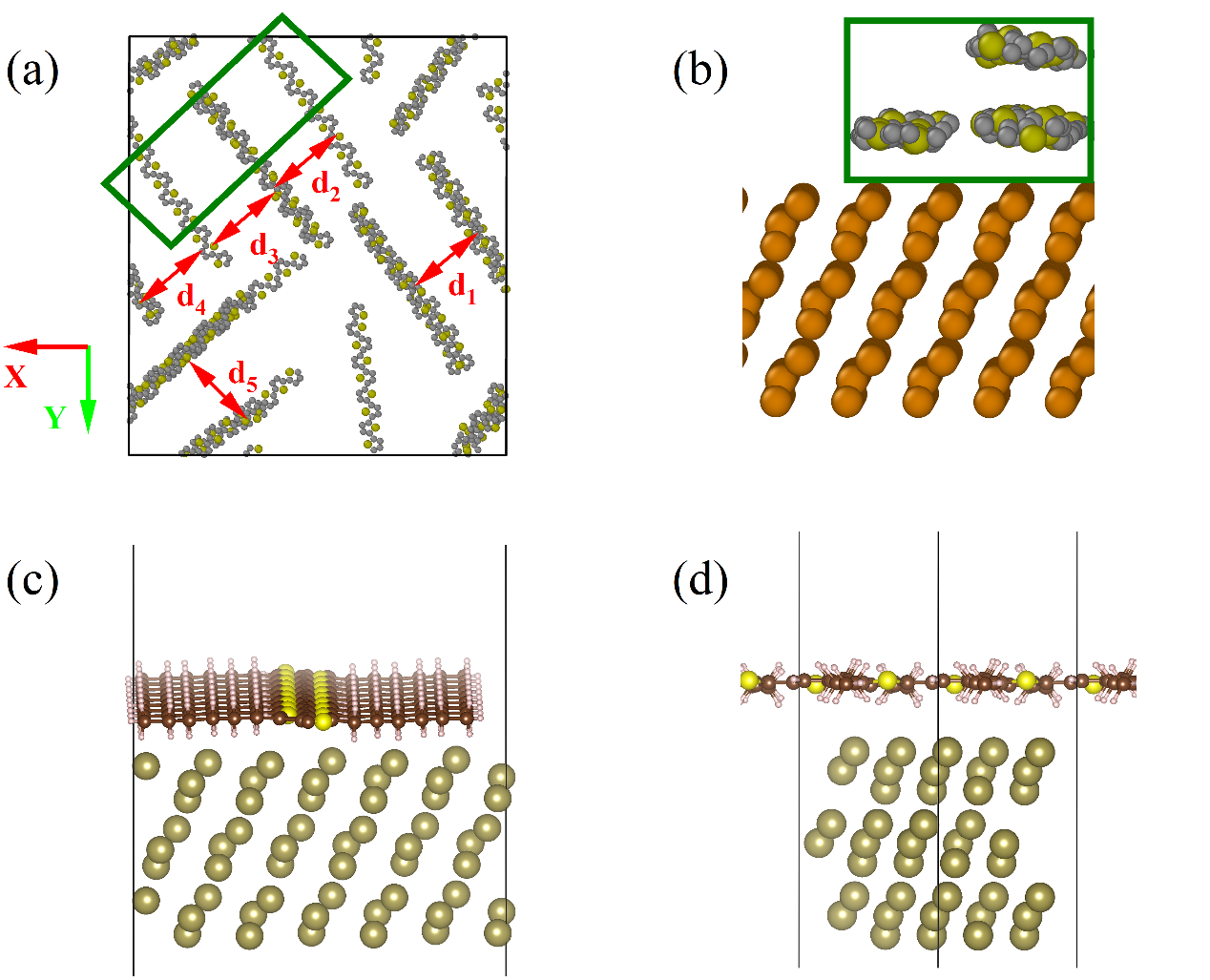


Fig. S17. (a) The distribution of the backbones of the (3HT)_12_ oligomers in the XY plane in the final state of the MD simulation, where the distance distribution between different chains is represented by d_1_, d_2_, d_3_, d_4_, and d_5_. (b) Side view of the green region along the direction of the backbone. (c) The DFT structure constructed based on the MD results, showing the orientation of the P3HT chains. (d) Side view from the direction perpendicular to the backbone of the P3HT chains.


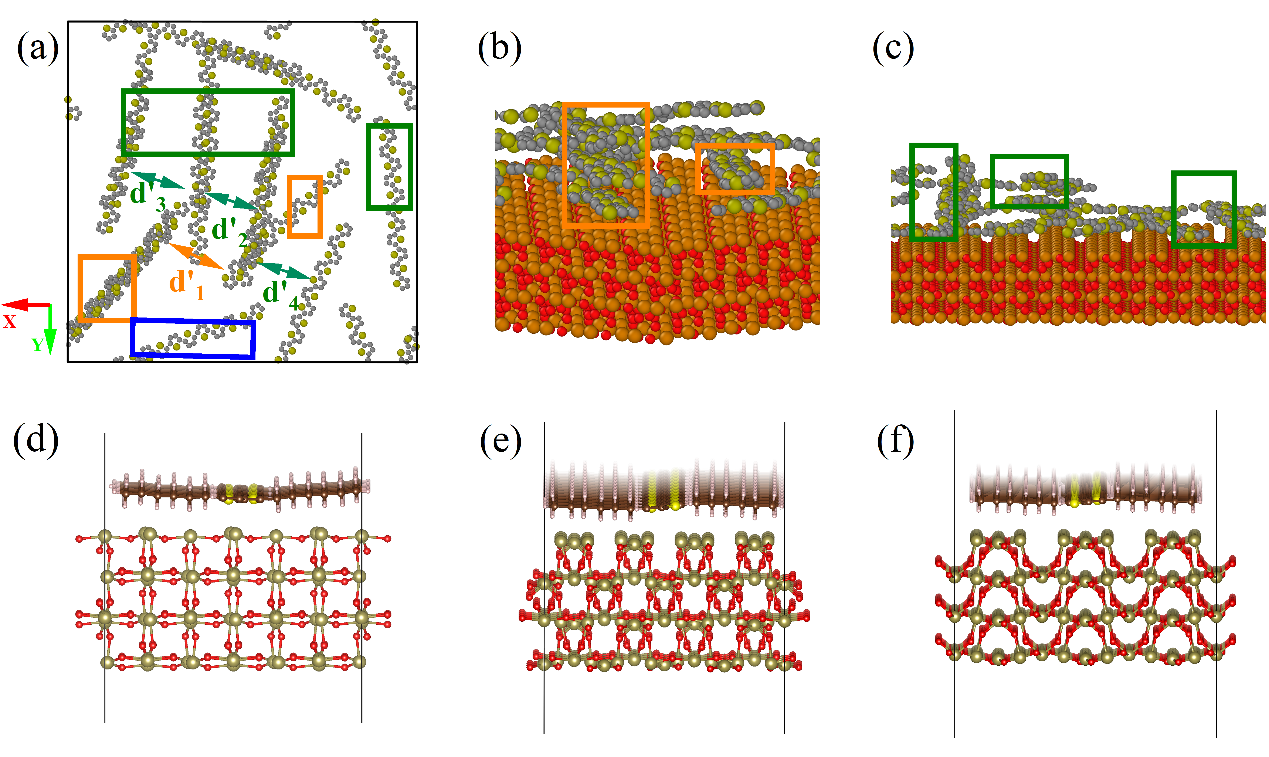


Fig. S18. (a) The distribution of the backbones of the (3HT)_12_ oligomers in the XY plane in the final state of the MD simulation, where the distance distribution between different chains is represented by d'_1_, d'_2_, d'_3_ and d'_4_. (b) Side views along the directions of the backbones of the (3HT)_12_ oligomers in the green region, corresponding to 0° with the Y-axis. (c), (d), (e), & (f) The DFT structures constructed corresponding to the preferential orientations of the backbones of the (3HT)_12_ oligomers (0°, 90°, 50°, and -40° with the Y-axis, respectively).

(b)


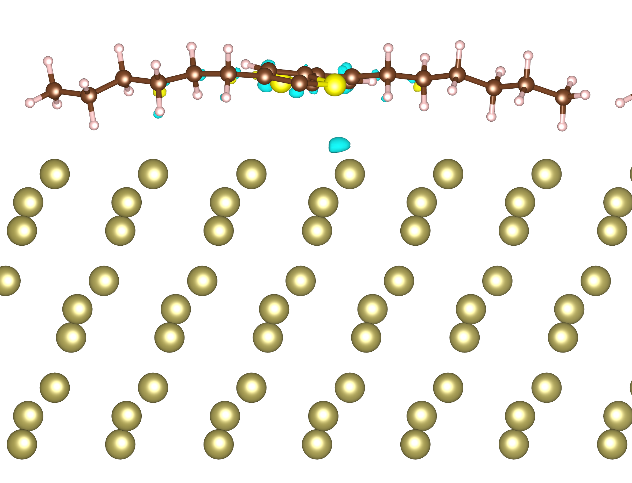

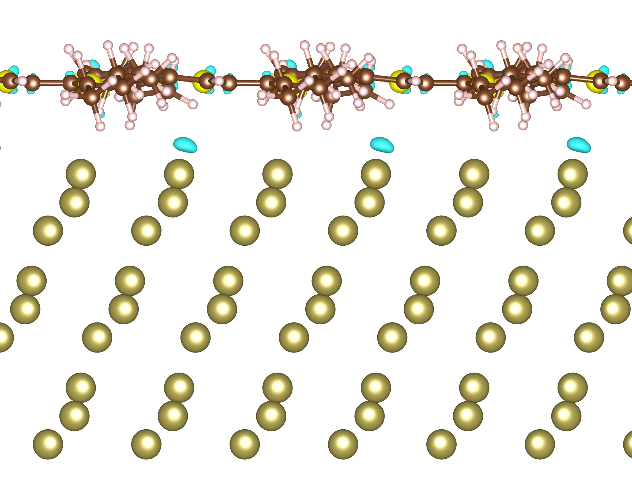


(a)


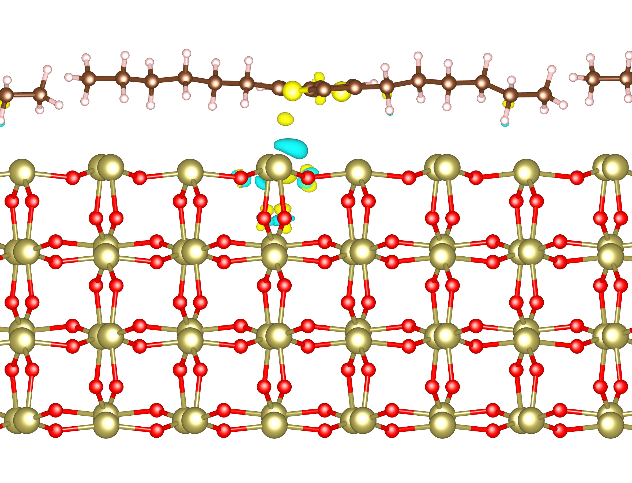

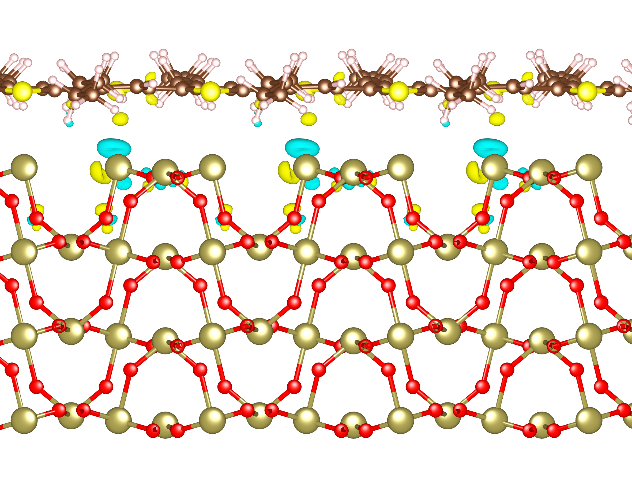


(d)

(c)

Fig. S19. The charge density difference distribution is illustrated in (a) and (b) for the P3HT/Te model and in (c) and (d) for the P3HT/TeO_2_ (90°) model. Subfigures (a) and (c) provide views along the backbone direction, while subfigures (b) and (d) offer views perpendicular to the backbone direction.


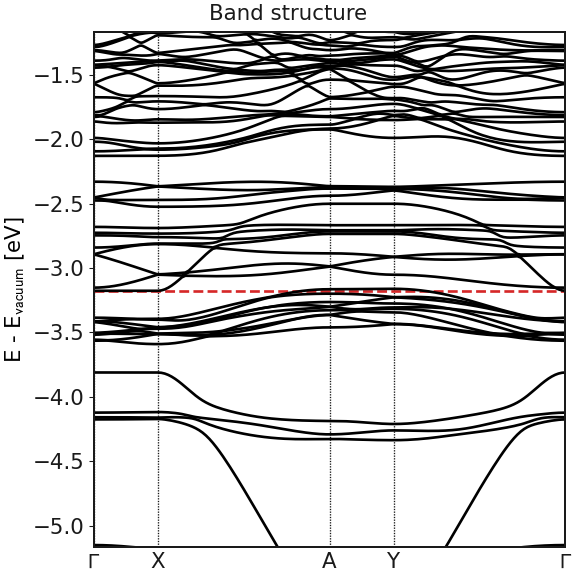

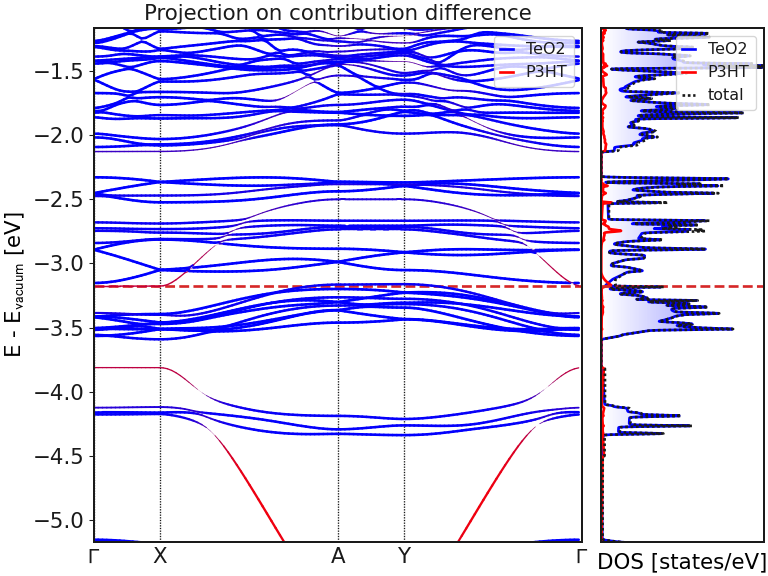


Fig. S20. The band structure (left) and the contribution projected band structure and density of state (right) of P3HT/TeO_2_ (90°) model. The red dashed line denotes the position of the Fermi level, while the energy reference is set with respect to the upper vacuum potential.


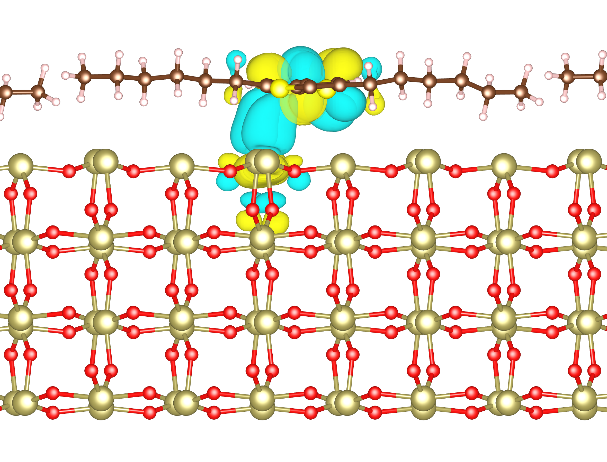

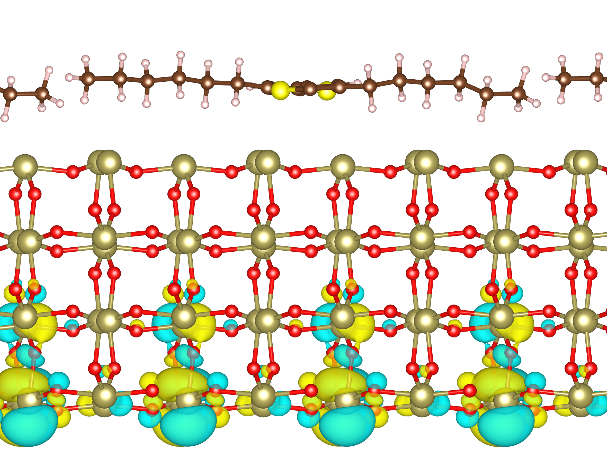


Fig. S21. HOMO (left) and LUMO (right) orbitals at Γ point in P3HT/TeO_2_ (90°) model.

## References

1. A. Zavabeti, P. Aukarasereenont, H. Tuohey, N. Syed, A. Jannat, A. Elbourne, K. A. Messalea, B. Y. Zhang, B. J. Murdoch, J. G. Partridge, M. Wurdack, *Nat. Electron.* 2021, **4**, 277.

2. C. Yan, B. H. Le, D. J. Kang, *J. Mater. Chem. A* 2014, **2**, 5394.

3. Z. Liu, T. Yamazaki, Y. Shen, T. Kikuta, N. Nakatani, *Jap. J. Appl. Phys.* 2008, **47**, 771.

4. A. P. Thompson, H. M. Aktulga, R. Berger, D. S. Bolintineanu, W. M. Brown, P. S. Crozier, P. J. in't Veld, A. Kohlmeyer, S. G. Moore, T. D. Nguyen, R. Shan, *Comp. Phys. Commun.* 2022, **271**, 108171.

5. H. Sun, *J. Physic. Chem. B* 1998, **102**, 7338.

6. T. S. Asche, P. Behrens, A. M. Schneider, *J. Sol-Gel Sci. & Tech.* 2017, **81**, 195.

7. N. A. Ran, S. Roland, J. A. Love, V. Savikhin, C. J. Takacs, Y. T. Fu, H. Li, V. Coropceanu, X. Liu, J. L. Brédas, G. C. Bazan. *Nat. Commun*. 2017, **8**, 79.

8. S. W. de Leeuw, J. W. Perram, E. R. Smith, *A. Math. Physic. Sci*. 1980, **373**, 27.

9. V. Blum, R. Gehrke, F. Hanke, P. Havu, V. Havu, X. Ren, K. Reuter, M. Scheffler, *Comp. Phys. Commun.* 2009, **180**, 2175.

10. J. P. Perdew, K. Burke, M. Ernzerhof, *Physic. Rev. Lett*. 1996, **77**, 3865.

11. A. Tkatchenko, M. Scheffler, *Physic. Rev. Lett*. 2009, **102**, 073005.

12. R. F. Bader, *Acc. Chem. Res*. 1985, **18**, 9.

13. W. Tang, E. Sanville, G. Henkelman, *J. Phys.Cond. Matt.* 2009, **21**, 084204.

14. M. Yu, D. R. Trinkle, *J. Chem. Phys.* 2011, **134**.

15. K. Momma, F. Izumi, *J. Appl. Crystallogr*. 2008, **41**, 653.

16. C. Poelking, K. Daoulas, A. Troisi, D. Andrienko, *Mol. Sca. Sol. Cell Dev*. 2014, **139**.

17. G. J. Snyder, E. S. Toberer, *Nature Mater.* 2008, **7**, 105.

18. M. D. L. Gonzalez-Juarez, M. A. Isaacs, D. Bradshaw, I. Nandhakumar, *ACS Appl. Mater. Interfaces* 2023, **15**, 5478.

19. Y. Lu, R. Liu, X. C. Hang, D. J. Young, *Poly. Chem.* 2021, **12**, 2115.

20. S. Qu, Q. Yao, L. Wang, Z. Chen, K. Xu, H. Zeng, W. Shi, T. Zhang, C. Uher, L. Chen, *NPG Asia Materials* 2016, **8**, 292.

21. X. Wu, Q. Tao, D. Li, Q. Wang, X. Zhang, H. Jin, J. Li, S. Wang, X. Xu, *Nano Research* 2021, **14**, 4725-31.
